# Variational Bayes for high-dimensional proportional hazards models with applications within gene expression

**DOI:** 10.1093/bioinformatics/btac416

**Published:** 2022-06-25

**Authors:** Michael Komodromos, Eric O Aboagye, Marina Evangelou, Sarah Filippi, Kolyan Ray

**Affiliations:** Department of Mathematics, Imperial College London, London SW7 2AZ, UK; Department of Surgery and Cancer, Imperial College London, London W12 0NN, UK; Department of Mathematics, Imperial College London, London SW7 2AZ, UK; Department of Mathematics, Imperial College London, London SW7 2AZ, UK; Department of Mathematics, Imperial College London, London SW7 2AZ, UK

## Abstract

**Motivation:**

Few Bayesian methods for analyzing high-dimensional sparse survival data provide scalable variable selection, effect estimation and uncertainty quantification. Such methods often either sacrifice uncertainty quantification by computing maximum *a posteriori* estimates, or quantify the uncertainty at high (unscalable) computational expense.

**Results:**

We bridge this gap and develop an interpretable and scalable Bayesian proportional hazards model for prediction and variable selection, referred to as sparse variational Bayes. Our method, based on a mean-field variational approximation, overcomes the high computational cost of Markov chain Monte Carlo, whilst retaining useful features, providing a posterior distribution for the parameters and offering a natural mechanism for variable selection via posterior inclusion probabilities. The performance of our proposed method is assessed via extensive simulations and compared against other state-of-the-art Bayesian variable selection methods, demonstrating comparable or better performance. Finally, we demonstrate how the proposed method can be used for variable selection on two transcriptomic datasets with censored survival outcomes, and how the uncertainty quantification offered by our method can be used to provide an interpretable assessment of patient risk.

**Availability and implementation:**

our method has been implemented as a freely available R package survival.svb (https://github.com/mkomod/survival.svb).

**Supplementary information:**

Supplementary data are available at *Bioinformatics online*.

## 1 Introduction

The development of high-throughput sequencing technologies has led to the production of large-scale molecular profiling data, allowing us to gain insights into underlying biological processes (Widłak, 2013). One such technology is microarray sequencing, in which mRNA counts are used to describe gene expression. Such data, known as transcriptomics, are widely used in the biomedical domain and when analyzed alongside survival times have provided extraordinary opportunities for biomarker characterization and prognostic modeling (Bøvelstad *et al.*, 2007; Lightbody *et al.*, 2019; Lloyd *et al.*, 2015; Lu *et al.*, 2021). However, profiling data are often high-dimensional, which introduces several statistical challenges including: (i) variable selection, (ii) effect estimation of the features, (iii) uncertainty quantification and (iv) scalable computation. The task of variable selection is particularly important, as few genes typically have an effect on the outcome. Motivated by clinical applicability, we propose a state-of-the-art scalable (variational) Bayesian variable selection method for the proportional hazards model (PHM).

In recent years, several methods have been proposed to analyze sparse high-dimensional data, with one of the most popular being the LASSO (Tibshirani, 1996). As biomedical studies are often concerned with clinical phenotypes, such as time to disease recurrence or overall survival time, these methods have been adapted to support survival analysis (Antoniadis *et al.*, 2010; Witten and Tibshirani, 2010). For instance, the LASSO, ridge and elastic-net penalties have all been extended to the PHM (Gui and Li, 2005; Simon *et al.*, 2011; Tibshirani, 1997; Zou and Hastie, 2005). More recently, Bayesian shrinkage and variable selection methods have grown in popularity (Bai *et al.*, 2021; Bhadra *et al.*, 2019; Carvalho *et al.*, 2010; Lewin *et al.*, 2019; Li and Zhang, 2010; O’Hara and Sillanpää, 2009; Park and Casella, 2008), with several methods being extended to survival data (Maity *et al.*, 2020; Nikooienejad *et al.*, 2020; Tang *et al.*, 2017).

Bayesian approaches to variable selection are popular, not least since the relevance of a covariate can be assessed simply by computing the posterior probability that it is included in a model. This recasts variable selection as a model selection problem (George and McCulloch, 1993; Mitchell and Beauchamp, 1988), with every possible model assigned an individual posterior probability. One of the most popular such model selection priors is the spike-and-slab prior, see (Banerjee *et al.*, 2021) for a recent survey. However, exact posterior computation involves summing over $2^{p}$ models, where *p* is the number of covariates, which is intractable for even moderate *p*. Markov chain Monte Carlo (MCMC) methods avoid this problem but are known to have difficulty efficiently exploring the model space for high-dimensional covariates (Ormerod *et al.*, 2017), especially for the problem sizes found in many modern omics studies which motivate our work. This high computational cost has led to several methods either making continuous relaxations, giving rise to *continuous shrinkage priors* (Banerjee *et al.*, 2021; O’Hara and Sillanpää, 2009), or computing only *maximum a posteriori* (MAP) estimates, thereby not offering the full Bayesian machinery. Since we wish to preserve certain interpretable features arising from the original discrete model selection approach, such as inclusion probabilities of particular covariates for variable selection, we instead turn to variational inference.

Variational inference (VI) is a popular scalable approximation technique, which has proven to be an effective tool for approximate Bayesian inference in many settings. VI involves minimizing the Kullback–Leibler divergence between a family of tractable distributions, called the *variational family*, and the posterior distribution; thereby recasting conditional inference as an optimization problem. The resulting minimizer is then used for downstream Bayesian inference. Though the approximation does not provide exact Bayesian inference, computationally convenient variational families can dramatically increase scalability. A common choice being *mean-field families*, under which the model parameters are independent. For a detailed review of VI, we direct the reader to Blei *et al.* (2017) and Zhang *et al.* (2019).

We propose a scalable and interpretable Bayesian PHM using a sparsity-inducing spike-and-slab prior with Laplace slab and Dirac spike, referred to as *sparse variational Bayes* (**SVB**). Since the posterior is computationally intractable, we use a mean-field variational approximation based on a factorizable family of spike-and-slab distributions, thereby preserving certain desirable discrete model selection aspects while providing scalable approximate Bayesian inference. We derive a coordinate-ascent algorithm for our implementation and investigate its performance in extensive simulations, comparing it against the posterior obtained via MCMC and demonstrating that the variational Bayes posterior can be used as a viable alternative, whilst being orders of magnitude faster to compute. We further compare with other state-of-the-art Bayesian variable selection methods, demonstrating comparable or better performance in many settings. Finally, we analyze two transcriptomic datasets involving ovarian and breast cancer data with censored survival outcomes, yielding biologically interpretable results.

Various versions of this sparse variational family have been employed in linear and logistic regression models (Carbonetto and Stephens, 2012; Logsdon *et al.*, 2010; Ormerod *et al.*, 2017; Ray *et al.*, 2020; Ray and Szabó, 2021; Titsias and Lázaro-Gredilla, 2011) with some of these works specifically motivated by high-dimensional genomic applications. While most of these works use Gaussian distributions for the slab component, we instead follow Ray and Szabó (2021) in using a Laplace prior slab since Gaussian prior slabs are known to cause excessive shrinkage leading to potentially poor performance, even when exact posterior computation is possible (Castillo and van der Vaart, 2012). Our work can thus be viewed as extending ideas from the sparse VI literature to the setting of survival analysis under censoring.

More generally, (not necessarily sparse) VI has proven to be an effective tool for approximate Bayesian inference and has seen wide use in several settings, including linear and logistic regression (Jaakkola and Jordan, 1996; Knowles and Minka, 2011), group factor analysis (Klami *et al.*, 2015), topic modeling (Blei and Lafferty, 2007), clustering (Teschendorff *et al.*, 2005) and Gaussian processes (Opper and Arachambeau, 2009) amongst others, with many of these methods employed in genomic and transcriptomic studies (Logsdon *et al.*, 2010; Papastamoulis *et al.*, 2014; Svensson *et al.*, 2020; Zhang and Flaherty, 2017).

## 2 Materials and methods

**Notation:** Let $D={\left\{(t_{i},\delta_{i},x_{i})\right\}}_{i=1}^{n}$ denote the observed data, where $t_{i}\in R^{+}$ is an observed (possibly right censored) survival time, $\delta_{i}\in \{0,1\}$ is a censoring indicator with $\delta_{i}=0$ if the observation is right censored and $\delta_{i}=1$ if the observation is uncensored, and $x_{i}={\left(x_{i1},\ldots ,x_{ip}\right)}^{⊤}\in R^{p}$ is a vector of explanatory variables.

### 2.1 Survival analysis and the proportional hazards model

Let T denote a random variable for an event time with density *f*(*t*) and cumulative distribution function *F*(*t*). Then the *survival function*, the probability a subject survives past time *t*, is given by

(1) $$S(t)=1-F(t)=\text{exp}\;\left(-\int_{0}^{t}h(s)ds\right)=\text{exp}\;\left(-H(t)\right),$$

where $H(t)=\int_{0}^{t}h(s)ds$ is the cumulative hazard rate and h(t)=f(t)/S(t) is the *hazard rate*, the instantaneous rate of failure at time *t*. Importantly, expressing *S*(*t*) in terms of the hazard function *h* provides a natural mechanism for analyzing survival times by assuming a form for *h* (Clark *et al.*, 2003; Ibrahim *et al.*, 2001).

One such form, used to quantify the effect of features collected alongside survival times, is the *proportional hazards model*, wherein,

(2) $$h(t;x,\beta )=h_{0}(t)\;\text{exp}\;\left(\beta^{⊤}x\right),$$

where $h_{0}(t)$ is a baseline hazard rate and $\beta ={\left(\beta_{1},\ldots ,\beta_{p}\right)}^{⊤}\in R^{p}$ are the model coefficients corresponding to the potential covariates of interest. Typically, estimating *β* is done by maximizing the *partial likelihood*,

(3) $$L_{p}(D;\beta )=\prod_{\left\{i:\delta_{i}=1\right\}}\frac{\;\text{exp}\;\left(\beta^{⊤}x_{i}\right)}{\sum_{r\in R(t_{i})}\;\text{exp}\;\left(\beta^{⊤}x_{r}\right)},$$

where $R(t_{i})=\{r:t_{r}\geq t_{i}\}$ (Cox, 1972, 1975). Under the partial likelihood, the baseline hazard rate $h_{0}(t)$ is treated as a nuisance parameter and not specified, meaning the survival function is not directly accessible without further assumptions on the hazard rate. This approach is commonly used when the main interest is on quantifying the effect of covariates on the survival time to understand the underlying mechanisms, rather than purely for predictive purposes. Since our focus is on variable selection and analyzing effect sizes, we use the partial likelihood to compute the posterior.

The use of the partial likelihood (3) is common in Bayesian survival analysis and can be understood via multiple Bayesian and frequentist justifications (Ibrahim *et al.*, 2001). For the frequentist, the partial likelihood is the empirical likelihood with the maximum likelihood estimator (MLE) for the cumulative baseline hazard function *H*_0_ plugged in, i.e. the profile likelihood (Murphy and Van Der Vaart, 2000). Using it in a Bayesian way thus means we are fitting a prior to our parameter of interest *β* and an MLE on the nuisance parameter *H*_0_. For the Bayesian, assigning a Gamma process prior to *H*_0_, marginalizing the posterior over *H*_0_ and taking the limit as the prior on *H*_0_ becomes non-informative, gives a marginal posterior for *β* exactly based on the partial likelihood (3) (Kalbfleisch, 1978). Thus using (3) can be viewed as using a diffuse Gamma process prior on the nuisance parameter *H*_0_.

### 2.2 Prior and variational family

We consider a spike-and-slab prior (George and McCulloch, 1993; Mitchell and Beauchamp, 1988) for the model coefficients *β*. Our choice of prior is conceptually natural for variable selection problems as it leads to interpretable inference regarding the inclusion probabilities of individual features. However, unlike the original formulation which uses Gaussian slabs, we use Laplace slabs, since Gaussian slabs are known to overly shrink the true large signals (Castillo and van der Vaart, 2012; Ray and Szabó, 2021). Formally, the prior distribution, Π(β,z,w), has hierarchical representation,

(4) $$\begin{array}{c} \beta_{j}|z_{j}\;\overset{\text{ind}}{∼}\;\;z_{j}\text{Laplace}(\lambda )+(1-z_{j})\delta_{0}\\ z_{j}|w_{j}\;\overset{\text{ind}}{∼}\;\;\text{Bernoulli}(w_{j})\\ w_{j}\;\overset{\text{iid}}{∼}\;\;\text{Beta}(a_{0},b_{0}), \end{array}$$

where $\delta_{0}$ is a Dirac mass at zero, Laplace(*λ*) has density function $\frac{\lambda }{2}e^{-\lambda |x|}$ on R and $\lambda ,a_{0},b_{0}>0$. Placing a hyperprior on $\left(w_{j}\right)$ allows mixing over the sparsity level and allows adaptation to the unknown sparsity. The posterior density is proportional to the partial likelihood *L_p_* in (3) times the joint prior density, formally,

(5) $$\pi (\beta ,z,w|D)\propto L_{p}(D;\beta )\pi (\beta ,z,w),$$

where $\beta ={\left(\beta_{1},\ldots ,\beta_{p}\right)}^{⊤}\in R^{p},\;z={\left(z_{1},\ldots ,z_{p}\right)}^{⊤}\in {\left\{0,1\right\}}^{p}$ and $w={\left(w_{1},\ldots ,w_{p}\right)}^{⊤}\in {\left[0,1\right]}^{p}$.

Since the posterior (5) is computationally intractable, we use a variational approximation. For the variational family, we choose a mean-field family given by the product of independent spike-and-slab distributions with normal slab and Dirac spike for each coefficient:

(6) $$Q=\left\{Q_{\mu ,\sigma ,\gamma }=\overset{p}{\underset{j=1}{⊗}}\left[\gamma_{j}N(\mu_{j},\sigma_{j}^{2})+(1-\gamma_{j})\delta_{0}\right]\right\},$$

where $\mu_{j}\in R,\sigma_{j}\in R^{+},\gamma_{j}\in [0,1]$. The notation ⊗ means a product measure implying coordinate independence, so that $\beta ∼Q_{\mu ,\sigma ,\gamma }$ means

$$\beta_{j}\overset{\text{ind}}{∼}\gamma_{j}N(\mu_{j},\sigma_{j}^{2})+(1-\gamma_{j})\delta_{0}.$$

Our choice of Q thereby provides scalability and maintains the property of variable selection via the Dirac mass, since the quantities $\gamma_{j}=Q(\beta_{j}\neq 0)$ are the inclusion probabilities. The variational posterior is then given by finding an element Q∈Q minimizing the KL divergence between *Q* and the posterior distribution Π(·|D),

(7) $$\tilde{\Pi }=\underset{Q_{\mu ,\sigma ,\gamma }\in Q}{\text{argmin}}\text{KL}\left(Q_{\mu ,\sigma ,\gamma }\;||\;\Pi (\cdot |D)\right),$$

which is then used for inference. Note this approximation has *O*(*p*) parameters compared to the full posterior dimension $O(2^{p})$. As with all mean-field approximation, dependent information between the components of *β* are lost, such as whether two coefficients *β_i_* and *β_j_* are likely to be selected simultaneously or not.

### 2.3 Coordinate-ascent algorithm

A convenient method for computing the mean-field variational posterior $\tilde{\Pi }$ is coordinate-ascent variational inference (CAVI) (Blei *et al.*, 2017). In CAVI, the parameters $\mu_{j},\sigma_{j},\gamma_{j}$ for j=1,…,p are sequentially updated by finding the values that minimize the KL divergence between the variational family and the posterior, whilst all other parameters are kept fixed, iterating until convergence. This reduces the overall optimization problem to a sequence of one-dimensional optimization problems.

Minimizing the objective (7) is intractable for the Bayesian PHM due to the form of likelihood (3) and so we instead minimize an upper bound for the KL divergence. Such surrogate type functionals are well-used in variational inference, for example in logistic regression (Depraetere and Vandebroek, 2017; Jaakkola and Jordan, 1996; Knowles and Minka, 2011) and can lead to an increase in accuracy.

The component-wise variational updates for *μ_j_* and *σ_j_* are given by the minimizers of

(8) $$\begin{array}{c} f(\mu_{j};\mu_{-j},\sigma ,\gamma ,z_{j}=1)=\\ \;\sum_{\left\{i:\delta_{i}=1\right\}}\left(\text{log}\;\sum_{r\in R(t_{i})}M(x_{rj},\mu_{j},\sigma_{j})P_{j}(x_{r},\mu ,\sigma ,\gamma )-\mu_{j}x_{ij}\right)\\ \;+\lambda \sigma_{j}\sqrt{2/\pi }e^{-\mu_{j}^{2}/(2\sigma_{j}^{2})}+\lambda \mu_{j}(1-2\Phi (-\mu_{j}/\sigma_{j})) \end{array}$$

and

(9) $$\begin{array}{c} g(\sigma_{j};\mu ,\sigma_{-j},\gamma ,z_{j}=1)=\\ \;\sum_{\left\{i:\delta_{i}=1\right\}}\left(\text{log}\;\sum_{r\in R(t_{i})}M(x_{rj},\mu_{j},\sigma_{j})P_{j}(x_{r},\mu ,\sigma ,\gamma )\right)\\ \;+\lambda \sigma_{j}\sqrt{2/\pi }e^{-\mu_{j}^{2}/(2\sigma_{j}^{2})}+\lambda \mu_{j}(1-2\Phi (-\mu_{j}/\sigma_{j}))-\text{log}\;\sigma_{j} \end{array}$$

where $M(x_{rj},\mu_{j},\sigma_{j})=\text{exp}(\mu_{j}x_{rj}+\frac{1}{2}\sigma_{j}^{2}x_{rj}^{2}),\;P_{j}(x_{r},\mu ,\sigma ,\gamma )=\prod_{k\neq j}\left(\gamma_{k}M(x_{rk},\mu_{k},\sigma_{k})+(1-\gamma_{k})\right)$ and Φ denotes the CDF of the standard normal distribution. The minimizers of these expressions do not have closed-form solutions, and therefore optimization routines are needed to find them, for instance via Brent’s method (Brent, 1973). Finally, the component-wise variational update for *γ_j_* is given by solving,

(10) $$\begin{array}{c} \;\text{log}\;\frac{\gamma_{j}}{1-\gamma_{j}}=\text{log}\;\frac{a_{0}}{b_{0}}-(\lambda \sigma_{j}\sqrt{2/\pi }e^{-\frac{\mu_{j}^{2}}{2\sigma_{j}^{2}}}+\lambda \mu_{j}(1-2\Phi (-\frac{\mu_{j}}{\sigma_{j}}))\\ +\sum_{\left\{i:\delta_{i}=1\right\}}(\;\text{log}\;\sum_{r\in R(t_{i})}M(x_{rj},\mu_{j},\sigma_{j})P_{j}(x_{r},\mu ,\sigma ,\gamma )\\ -\text{log}\;\sum_{r\in R(t_{i})}P_{j}(x_{r},\mu ,\sigma ,\gamma )-\mu_{j}x_{ij})+\text{log}\;\frac{\sqrt{2}}{\sqrt{\pi }\sigma_{j}\lambda })+\frac{1}{2} \end{array}$$

A full derivation of these expressions is provided in Supplementary Section SA.

Algorithm 1 summarizes the CAVI algorithm. We denote the RHS of (10) by $\zeta (\gamma_{j};\mu ,\sigma ,\gamma_{-j})$, and assess convergence by computing the change in μ,σ and *γ* after each iteration, stopping when the total absolute change is below a specified threshold (e.g. $10^{-3}$). While the evidence lower bound (ELBO) is often used to assess convergence, the ELBO is not analytically tractable in the present setting, which instead requires computationally expensive Monte Carlo integration to evaluate it. For this reason, we instead choose to assess convergence using the absolute change in μ,σ and *γ*.

Due to the non-convex objective in (7), CAVI generally only guarantees convergence to a local optimum, and therefore can be sensitive to initialization (Blei *et al.*, 2017). We found this to be the case for our method, particularly for *μ* and *γ*, therefore providing good starting values is generally important (see Supplementary Section SD for more details). In turn, we initialized *μ* using the LASSO with a small regularization hyperparameter, since *μ* corresponds to the unshrunk means if the variables are included in the model, and *γ* as ${\left(0.5,\ldots ,0.5\right)}^{⊤}$, since this corresponds to an initial inclusion probability of 0.5 for each feature. We found the proposed method is less sensitive to initial value of *σ*, for example initializing *σ* as ${\left(0.05,\ldots ,0.05\right)}^{⊤}$ is sufficient.

**Algorithm 1:** CAVI for VB approximation to posterior (5) 1: **require**  $D,\lambda ,a_{0},b_{0}$

2: Initialize μ,σ,γ

3: **while** not converged

4:    **for**  j=1,…,p

5:      $\mu_{j}\leftarrow {\text{argmin}}_{\mu_{j}\in R}\;\;f(\mu_{j};\mu_{-j},\sigma ,\gamma ,z_{j}=1)$// (8)

6:      $\sigma_{j}\leftarrow {\text{argmin}}_{\sigma_{j}\in R^{+}}\;g(\sigma_{j};\mu ,\sigma_{-j},\gamma ,z_{j}=1)$// (9)

7:      $\gamma_{j}\leftarrow \text{sigmoid}\;\zeta (\gamma_{j};\mu ,\sigma ,\gamma_{-j})$// (10)

8: **return**  μ,σ,γ.

### 2.4 Parameter tuning

The proposed method involves three prior parameters $\lambda ,a_{0}$ and *b*_0_ defined in (4), where *λ* controls the shrinkage imposed on $\beta_{j}|z_{j}=1$, with large values imposing more shrinkage, and *a*_0_ and *b*_0_ control the shape of the Beta distribution, whose expectation $a_{0}/(a_{0}+b_{0})$ reflects the *a priori* proportion of non-zero coefficients. Generally, our method is not particularly sensitive to the prior parameters (see Supplementary Section SD for a numerical investigation) and in practice using sensible *a priori* choices is appropriate for most settings. For example, if it is believed there are a small number of non-zero coefficients with moderate effect sizes, taking *a*_0_ as a small constant (such as 1,10,p/100), $b_{0}=p$ and *λ* between 0.5 and 2.0 is appropriate.

If an *a priori* choice is unavailable, the prior parameters can be tuned using the data. To do so, we suggest performing a grid search over a predefined set of values, selecting the element that maximizes a given goodness of fit measure, several options of which are presented in Supplementary Section SB. Furthermore, when tuning *a*_0_ and *b*_0_, to limit computation we suggest fixing *b*_0_ and searching across a set of values for *a*_0_, thereby exploring different values of the *a priori* inclusion probability.

### 2.5 Implementation

A freely available implementation is available for the R programming language via the package survival.svb, with functions available for fitting and evaluating models.

## 3 Simulation study

We use simulations to validate the proposed method, referred to as **SVB**. Firstly, we compare the variational posterior to the posterior obtained via MCMC, assessing whether our approximation can be used as a viable alternative. Secondly, we compare against other state-of-the-art Bayesian variable selection methods for the PHM. R scripts to reproduce our results can be found at https://github.com/mkomod/svb.exp.

### 3.1 Simulation design

Data are simulated for i=1,…,n observations, each having a survival time *t_i_*, censoring indicator *δ_i_* and *p* continuous predictors $x_{i}\in R^{p}$. The survival time is sampled independently from $T\;|\;x_{i},\beta_{0},h_{0}$, which has density $f(t;x,\beta_{0},h_{0})=h_{0}(t)\;\text{exp}\;\left(\beta_{0}^{⊤}x-e^{\beta_{0}^{⊤}x}\int_{0}^{t}h_{0}(s)ds\right)$, where we have taken $h_{0}(t)=1$ and where the coefficient vector $\beta_{0}\in R^{p}$ contains *s* non-zero elements with values sampled iid. uniformly from [−2.0,−0.5]∪[0.5,2.0] and indices chosen uniformly at random. To introduce censoring, we sample $d_{i}\overset{\text{iid}}{∼}U(0,1)$, letting $\delta_{i}=I(d_{i}>c)$ where c∈[0,1] is the censoring proportion, and set $t_{i}\leftarrow t\prime_{i}$ where $t\prime_{i}\overset{\text{ind}}{∼}U(0,t_{i})$ if $\delta_{i}=0$, leaving *t_i_* unchanged otherwise. Finally, the predictors are generated from one of four different settings designed to examine the behavior under varying degrees of difficulty:

- *Setting 1*, an independent setting where $x_{i}\overset{\text{iid}}{∼}N(0_{p},I_{p})$.
- *Setting 2*, a fairly challenging setting where predictors are moderately correlated within groups and independent between groups, formally $x_{i}\overset{\text{iid}}{∼}N(0,\Sigma )$ with $\text{diag}(\Sigma )=1,\;\Sigma_{ij}=0.6$ for $i\neq j,\;i,j=50k+1,\ldots ,50(k+1),\;k=0,\ldots ,p/50-1,\;\Sigma_{ij}=0$ otherwise. The setting is similar to Tang *et al.* (2017).
- *Setting 3*, a challenging setting where $x_{i}\overset{\text{iid}}{∼}N(\mu ,\Sigma )$ with μ,Σ estimated from the design of the TCGA dataset analyzed in Section 4.1. The *s* causal variables are randomly selected to correspond to features with a variance of at least 1.0.
- *Setting 4*, a realistic setting where the first *p* predictors are taken from the TCGA dataset analyzed in Section 4.1 and the *s* causal features are selected as in Setting 3.

To evaluate the methods, we examine the accuracy of the corresponding point estimates, quality of the variables selected, and (if applicable) the uncertainty quantification. The point estimates are assessed via the $\ell_{2}-\text{error},\;||\beta_{0}-\hat{\beta }||$ and the $\ell_{1}-\text{error},\;|\beta_{0}-\hat{\beta }|$, where $\hat{\beta }$ is either a MAP estimate for *β* or the posterior mean if a distribution is available. For the variables selected the: (i) true positive rate (TPR) (ii) false discovery rate (FDR) and (iii) area under the curve (AUC) of the receiver operator characteristic curve are computed. For the TPR and FDR a coefficient is considered to have been selected if the posterior inclusion probability is at least 0.5. Finally, regarding uncertainty quantification, we evaluate the marginal credible sets by computing the: (i) empirical coverage, i.e. the proportion of times the true coefficient $\beta_{0,j}$ is contained in the credible set, and (ii) set size, given by the Lebesgue measure of the set. Details regarding the construction of the credible sets are presented where appropriate. For all metrics, we report the median, 5% and 95% quantiles across 100 replicates unless otherwise stated.

### 3.2 Simulation results

#### 3.2.1 Comparison to MCMC

To assess how well the variational posterior matches the target (computationally challenging) posterior from (5), we compare the performance of our approach against the approximate yet asymptotically exact posterior obtained via MCMC. To do so, data is generated as described in Section 3.1, taking (n,p,s)=(200,1000,10) and c∈{0.25,0.4}, where we have kept *n* and *p* small so we can run our MCMC sampler in a reasonable amount of time. The MCMC sampler (described in Supplementary Section SC.1) was run for 10,000 iterations with a burn-in period of 1,000 iterations. For both methods, we used prior parameters $\lambda =1,a_{0}=1$ and $b_{0}=p$. Results are presented in Table 1.

**Table 1.** Comparison of variational to MCMC posterior taking (n,p,s)=(200,1000,10) and c∈{0.25,0.4}, presented is the median and (5%,95%) quantiles

Setting	*c*	Method	$\ell_{2}$ -error	$\ell_{1}$ -error	TPR	FDR	AUC	Runtime
*Setting 1*	0.25	SVB	0.368 (0.21, 0.70)	1.000 (0.52, 1.86)	1.000 (0.90, 1.00)	0.000 (0.00, 0.00)	1.000 (1.00, 1.00)	18.5 s (13.5 s , 25.6 s)
		MCMC	0.412 (0.20, 0.75)	1.017 (0.48, 2.01)	1.000 (0.90, 1.00)	0.000 (0.00, 0.00)	1.000 (1.00, 1.00)	1 h 24 m (1 h 7 m , 1 h 50 m)
	0.4	SVB	0.428 (0.23, 0.89)	1.138 (0.63, 2.45)	1.000 (0.90, 1.00)	0.000 (0.00, 0.00)	1.000 (0.95, 1.00)	21.9 s (14.5 s , 30.5 s)
		MCMC	0.506 (0.26, 0.98)	1.300 (0.69, 2.74)	1.000 (0.80, 1.00)	0.000 (0.00, 0.00)	1.000 (1.00, 1.00)	1 h 28 m (1 h 25 m , 1 h 30 m)
*Setting 2*	0.25	SVB	0.376 (0.20, 0.73)	1.031 (0.58, 2.07)	1.000 (0.90, 1.00)	0.000 (0.00, 0.00)	1.000 (1.00, 1.00)	18.9 s (14.4 s , 25.4 s)
		MCMC	0.405 (0.21, 0.81)	1.059 (0.58, 2.18)	1.000 (0.90, 1.00)	0.000 (0.00, 0.00)	1.000 (1.00, 1.00)	1 h 14 m (1 h 6 m , 1 h 17 m)
	0.4	SVB	0.472 (0.23, 1.08)	1.176 (0.61, 2.96)	1.000 (0.90, 1.00)	0.000 (0.00, 0.00)	1.000 (0.95, 1.00)	24.0 s (17.3 s , 33.1 s)
		MCMC	0.520 (0.25, 1.08)	1.319 (0.62, 2.91)	1.000 (0.90, 1.00)	0.000 (0.00, 0.00)	1.000 (1.00, 1.00)	1 h 38 m (1 h 25 m , 2 h 4 m)
*Setting 3*	0.25	SVB	0.392 (0.18, 1.40)	1.079 (0.53, 3.28)	1.000 (0.90, 1.00)	0.000 (0.00, 0.09)	1.000 (0.95, 1.00)	29.2 s (16.9 s , 44.9 s)
		MCMC	0.418 (0.21, 1.01)	1.092 (0.54, 2.58)	1.000 (0.90, 1.00)	0.000 (0.00, 0.00)	1.000 (1.00, 1.00)	1 h 45 m (1 h 24 m , 1 h 49 m)
	0.4	SVB	0.470 (0.24, 1.57)	1.263 (0.63, 4.16)	1.000 (0.80, 1.00)	0.000 (0.00, 0.10)	1.000 (0.95, 1.00)	21.7 s (13.7 s , 33.2 s)
		MCMC	0.508 (0.23, 1.26)	1.236 (0.61, 3.45)	1.000 (0.80, 1.00)	0.000 (0.00, 0.09)	1.000 (1.00, 1.00)	1 h 36 m (1 h 30 m , 1 h 45 m)
*Setting 4*	0.25	SVB	0.393 (0.18, 1.12)	1.067 (0.50, 2.54)	1.000 (0.90, 1.00)	0.000 (0.00, 0.10)	1.000 (0.95, 1.00)	17.0 s (9.2 s , 24.9 s)
		MCMC	0.382 (0.17, 0.95)	1.007 (0.44, 2.47)	1.000 (0.90, 1.00)	0.000 (0.00, 0.10)	1.000 (1.00, 1.00)	1 h 5 m (1 h 3 m , 1 h 8 m)
	0.4	SVB	0.425 (0.18, 1.38)	1.171 (0.50, 2.85)	1.000 (0.90, 1.00)	0.000 (0.00, 0.10)	1.000 (0.95, 1.00)	25.8 s (14.8 s , 39.9 s)
		MCMC	0.486 (0.21, 1.13)	1.158 (0.53, 3.17)	1.000 (0.80, 1.00)	0.000 (0.00, 0.00)	1.000 (0.95, 1.00)	1 h 38 m (1 h 14 m , 1 h 46 m)

*Note*: Simulations were ran on Intel^®^ Xeon^®^ E5-2680 v4 2.40 GHz CPUs.

Regarding the point estimates, for both the MCMC and the variational posteriors we took $\hat{\beta }=({\hat{\beta }}_{1},\ldots ,{\hat{\beta }}_{p})\in R^{p}$ as the posterior mean, which for the latter is given by ${\hat{\beta }}_{j}=\gamma_{j}\mu_{j}$. Promisingly, both methods produce similar results, with near identical performance in all settings (Table 1). In particular, the similarity of the $\ell_{2}$-error and $\ell_{1}$-error suggests the posterior means are near identical. In terms of variable selection, both methods performed similarly. In particular, the TPR is comparable across the different settings, suggesting both methods are selecting a similar set of truly associated features. However, the upper quantile for the FDR is slightly larger for the variational posterior, meaning the MCMC posterior selects fewer spurious variables.

Finally, we examine the uncertainty quantification of each method via 95% marginal credible sets $S_{j},j=1,\ldots ,p$, which are given by: *S_j_* = *I_j_* if the posterior inclusion probability is greater than 0.95, $S_{j}=\{0\}$ if the posterior inclusion probability is <0.05, and $S_{j}=I_{j}\cup \{0\}$ otherwise, where *I_j_* is the smallest interval from the continuous component of our posterior such that *S_j_* contains 95% of the posterior mass. As expected, for the non-zero coefficients, the coverage of the MCMC posterior is slightly better than the coverage of the variational posterior (Table 2), meaning the credible sets of the variational posterior are sometimes not large enough to capture the true non-zero coefficients. This is further reflected by the smaller set sizes, highlighting the well-known fact that VI can underestimate the posterior variance (Blei *et al.*, 2017; Carbonetto and Stephens, 2012; Ray *et al.*, 2020; Zhang *et al.*, 2019). Promisingly, the coverage of the zero coefficients is equal to one for both methods, meaning the credible sets contain zero, and typically, as reflected by the set size, contain only zero.

**Table 2.** Coverage and set size for variational and MCMC posterior

Set.	*c*	Meth.	Cov. $\beta_{0}\neq 0$	Set size $\beta_{0}\neq 0$	Cov. $\beta_{0}=0$	Set size $\beta_{0}=0$
1	0.25	SVB	0.770 (0.202)	0.320 (0.013)	1.000 (0.000)	0.000 (0.000)
		MCMC	0.928 (0.138)	0.506 (0.039)	1.000 (0.000)	0.000 (0.000)
	0.4	SVB	0.774 (0.208)	0.355 (0.021)	1.000 (0.000)	0.000 (0.000)
		MCMC	0.914 (0.127)	0.570 (0.054)	1.000 (0.000)	0.000 (0.000)
2	0.25	SVB	0.703 (0.227)	0.306 (0.028)	1.000 (0.001)	0.000 (0.000)
		MCMC	0.904 (0.161)	0.522 (0.053)	1.000 (0.000)	0.000 (0.000)
	0.4	SVB	0.683 (0.262)	0.333 (0.039)	1.000 (0.001)	0.000 (0.000)
		MCMC	0.845 (0.218)	0.567 (0.101)	1.000 (0.000)	0.000 (0.000)
3	0.25	SVB	0.626 (0.288)	0.251 (0.020)	1.000 (0.000)	0.000 (0.000)
		MCMC	0.903 (0.140)	0.482 (0.047)	1.000 (0.000)	0.000 (0.000)
	0.4	SVB	0.619 (0.278)	0.276 (0.028)	1.000 (0.000)	0.000 (0.000)
		MCMC	0.873 (0.197)	0.540 (0.078)	1.000 (0.000)	0.000 (0.000)
4	0.25	SVB	0.672 (0.224)	0.252 (0.021)	1.000 (0.000)	0.000 (0.000)
		MCMC	0.921 (0.144)	0.483 (0.047)	1.000 (0.000)	0.000 (0.000)
	0.4	SVB	0.660 (0.249)	0.277 (0.025)	1.000 (0.001)	0.000 (0.000)
		MCMC	0.906 (0.156)	0.547 (0.059)	1.000 (0.000)	0.000 (0.000)

*Note*: Presented are means and std. dev.

Overall, the variational posterior displays similar performance to the MCMC posterior in key aspects for this setting with *p *=* *1000 and can be computed orders of magnitude faster (Table 1). Our results highlight that the variational posterior is particularly good at capturing the key features (posterior means and inclusion probabilities) and provides reasonable uncertainty quantification for individual features.

#### 3.2.2 Comparison to other methods

We perform a large-scale simulation study to empirically compare the performance of our method to two Bayesian variable selection methods. Within our study, data is generated as described in Section 3.1, taking (n,p,s)=(500,5000,30) and c∈{0.25,0.4} for all settings. Notably, under such a setting running MCMC would be computationally prohibitive, as highlighted in the previous section.

We compare against **BhGLM** (Tang *et al.*, 2017), a spike-and-slab LASSO method that uses a mixture of Laplace distributions with one acting as the spike and the other the slab, and **BVSNLP** (Nikooienejad *et al.*, 2020), which uses a mixture prior composed of a point mass at zero and an inverse moment prior. Notably, both **BhGLM** and **BVSNLP** use Cox’s partial likelihood in the posterior and return a MAP estimate for *β* as well as posterior inclusion probabilities for each feature. Finally, for each method we use the default hyperparameters and let *λ *= 1, $a_{0}=1$ and $b_{0}=p$ for **SVB**.

Generally, all methods produced excellent point estimates, with **SVB** obtaining the smallest median $\ell_{2}$-error and $\ell_{1}$-error in Settings 1 and 2, and **BhGLM** in Setting 4 (Table 3). Notably, **SVB** obtained the smallest lower (5%) quantile for the $\ell_{2}$-error and $\ell_{1}$-error in Settings 3 and 4, meaning the method can perform better than **BhGLM** but may be sensitive to the design matrix.

**Table 3.** Comparison of Bayesian variable selection methods, taking (n,p,s)=(500,5000,30) and c∈{0.25,0.4}, presented is the median and (5%,95%) quantiles

Setting	*c*	Method	$\ell_{2}$ -error	$\ell_{1}$ -error	TPR	FDR	AUC
*Setting 1*	0.25	SVB	0.378 (0.26, 0.89)	1.747 (1.16, 4.17)	1.000 (1.00, 1.00)	0.000 (0.00, 0.00)	1.000 (1.00, 1.00)
		BhGLM	1.206 (0.79, 1.78)	9.590 (7.22, 12.88)	1.000 (1.00, 1.00)	0.000 (0.00, 0.00)	1.000 (1.00, 1.00)
		BVSNLP	0.456 (0.33, 0.96)	2.007 (1.41, 4.57)	1.000 (1.00, 1.00)	0.000 (0.00, 0.03)	1.000 (1.00, 1.00)
	0.4	SVB	0.449 (0.31, 0.99)	2.056 (1.37, 4.87)	1.000 (1.00, 1.00)	0.000 (0.00, 0.00)	1.000 (1.00, 1.00)
		BhGLM	0.807 (0.53, 1.35)	6.458 (4.52, 9.31)	1.000 (1.00, 1.00)	0.000 (0.00, 0.00)	1.000 (1.00, 1.00)
		BVSNLP	0.518 (0.35, 1.44)	2.231 (1.52, 6.85)	1.000 (0.96, 1.00)	0.000 (0.00, 0.03)	1.000 (0.99, 1.00)
*Setting 2*	0.25	SVB	0.405 (0.29, 0.80)	1.823 (1.28, 3.78)	1.000 (1.00, 1.00)	0.000 (0.00, 0.00)	1.000 (1.00, 1.00)
		BhGLM	0.596 (0.45, 1.04)	4.494 (3.51, 6.89)	1.000 (1.00, 1.00)	0.000 (0.00, 0.00)	1.000 (1.00, 1.00)
		BVSNLP	0.475 (0.33, 0.90)	2.130 (1.47, 4.01)	1.000 (1.00, 1.00)	0.000 (0.00, 0.00)	1.000 (1.00, 1.00)
	0.4	SVB	0.491 (0.33, 1.05)	2.208 (1.45, 5.03)	1.000 (0.97, 1.00)	0.000 (0.00, 0.03)	1.000 (1.00, 1.00)
		BhGLM	0.551 (0.44, 0.86)	3.716 (2.98, 5.36)	1.000 (0.97, 1.00)	0.000 (0.00, 0.00)	1.000 (1.00, 1.00)
		BVSNLP	0.515 (0.37, 1.47)	2.238 (1.54, 6.71)	1.000 (1.00, 1.00)	0.000 (0.00, 0.00)	1.000 (1.00, 1.00)
*Setting 3*	0.25	SVB	1.040 (0.30, 3.17)	3.881 (1.36, 15.37)	0.967 (0.83, 1.00)	0.000 (0.00, 0.14)	0.983 (0.92, 1.00)
		BhGLM	0.590 (0.36, 1.57)	3.279 (2.23, 6.73)	1.000 (0.93, 1.00)	0.000 (0.00, 0.03)	1.000 (0.97, 1.00)
		BVSNLP	3.107 (1.74, 9.73)	12.262 (6.88, 47.67)	0.967 (0.53, 1.00)	0.000 (0.00, 0.53)	0.983 (0.78, 1.00)
	0.4	SVB	1.379 (0.36, 3.47)	5.728 (1.55, 17.47)	0.933 (0.77, 1.00)	0.033 (0.00, 0.13)	0.967 (0.88, 1.00)
		BhGLM	0.796 (0.41, 2.18)	4.035 (2.25, 10.92)	0.967 (0.87, 1.00)	0.000 (0.00, 0.07)	1.000 (0.95, 1.00)
		BVSNLP	3.867 (1.98, 11.44)	15.874 (7.99, 51.07)	0.967 (0.20, 1.00)	0.033 (0.00, 0.69)	0.983 (0.65, 1.00)
*Setting 4*	0.25	SVB	0.603 (0.29, 2.02)	2.298 (1.21, 8.84)	1.000 (0.90, 1.00)	0.000 (0.00, 0.08)	1.000 (0.95, 1.00)
		BhGLM	0.503 (0.35, 1.36)	3.141 (2.25, 5.59)	1.000 (0.93, 1.00)	0.000 (0.00, 0.03)	1.000 (0.97, 1.00)
		BVSNLP	2.946 (1.96, 8.72)	11.426 (6.98, 36.46)	1.000 (0.90, 1.00)	0.000 (0.00, 0.07)	1.000 (0.95, 1.00)
	0.4	SVB	1.092 (0.32, 2.83)	3.878 (1.40, 14.06)	0.967 (0.83, 1.00)	0.000 (0.00, 0.08)	0.983 (0.92, 1.00)
		BhGLM	0.674 (0.40, 1.64)	3.610 (2.28, 7.72)	1.000 (0.93, 1.00)	0.000 (0.00, 0.04)	1.000 (0.97, 1.00)
		BVSNLP	3.163 (2.14, 10.53)	12.227 (8.14, 45.64)	1.000 (0.73, 1.00)	0.000 (0.00, 0.32)	1.000 (0.87, 1.00)

Regarding the variables selected, all methods performed exceptionally well achieving the ideal values for the TPR, FDR and AUC in Settings 1 and 2 (Table 3). Within Settings 3, **BhGLM** obtained the best TPR, FDR and AUC closely followed by **SVB** and **BVSNLP**. Within Setting 4, all three methods obtained the ideal values when the censoring was low (*c *=* *0.25) and **BhGLM** performed best under moderate censoring (*c *=* *0.40). Further, **BhGLM** best controlled the FDR in Settings 3 and 4, obtaining the lowest upper (95%) quantile, closely followed by **SVB**. Finally, we note, **SVB** is the only method that provides uncertainty quantification, a direct application of which is demonstrated in Section 4.2.

## 4 Application

### 4.1 TCGA ovarian cancer data

The first dataset we analyzed is a transcriptomic dataset where the outcome of interest is overall survival. The dataset was collected from patients with ovarian cancer and has a sample size of *n *=* *580, of which 229 samples are right censored and 351 samples are uncensored, corresponding to a censoring rate of 39.5% (TCGA, 2022). Within the dataset there are p=12,042 covariates, which we pre-processed by removing features with a coefficient of variation below the median value (Mar *et al.*, 2011), leaving 6021 covariates which we centered before fitting our method.

When applying our method, we set $a_{0}=p/100$ and $b_{0}=p$, reflecting our prior belief that few genes are associated with the response. As we had no prior belief for *λ*, we performed 10-fold cross-validation to select the value, exploring a grid of values Λ={0.05, 0.1, 0.25,0.5, 0.75, 1.0, 1.25, 1.5,1.75, 2.0, 2.5, 3.0, 4.0,5.0}. To evaluate model fit we compute the: (i) ELBO$=E_{Q}\left[\text{log}\;L_{p}\right]-\text{KL}(Q||\Pi )$, (ii) expected log-likelihood under the variational posterior (ELL = $E_{Q}[\text{log}\;L_{p}(D;\beta )]$) and (iii) c-index, reporting the mean and standard deviation across the 10 folds for the training and validation sets in Supplementary Table S2. Notably, no single hyperparameter value stands out as being best, meaning the model is not particularly sensitive to the value of *λ*.

**Fig. 1.**
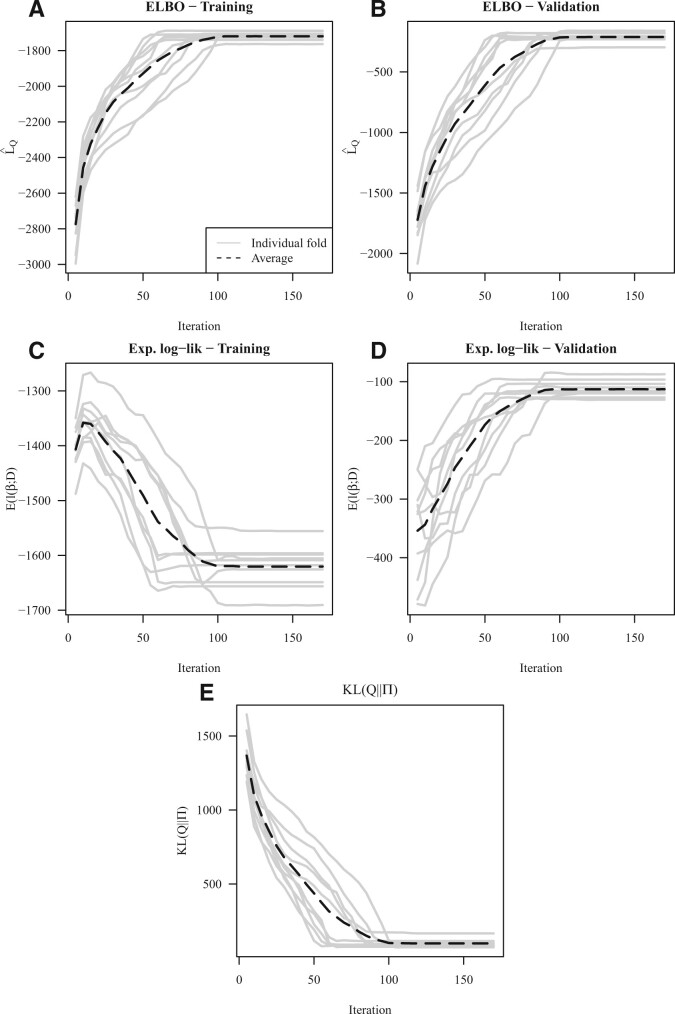
Ovarian cancer dataset model convergence diagnostics for *λ *= 1

To assess the model’s convergence diagnostics we examine the fit for *λ *= 1, and examine the change in: (i) ELBO, (ii) ELL and (iii) KL between the variational posterior and the prior, as we iterate our co-ordinate ascent algorithm (Fig. 1). Note the ELBO and ELL are computed for the training and validation sets, whereas KL(Q||Π) need only be computed for the training set. Notably, across the different folds the ELBO is increasing as the co-ordinate ascent algorithm is iterated (Fig. 1A and B), suggesting that the model fit is improving. Interestingly, the training ELL is decreasing (Fig. 1C), whereas the inverse is true for the validation ELL (Fig. 1D), meaning, initially the model is overfitting to the training data, and as we iterate begins to fit better to the unseen validation set. Further, the KL(Q||Π) is decreasing (Fig. 1E), therefore a greater degree of sparsity is enforced as we iterate, excluding more features and preserving the ones that best explain the variation in the response.

As we are using our model for variable selection, we examine the genes selected across the different values of *λ* and folds. Table 4 reports the names and selection proportion of genes, where the selection proportion is the number of times a particular gene has posterior inclusion probability greater than

(11) $$k^{*}=\underset{k\in [0,1]}{\text{argmax}}\left\{\frac{\sum_{j=1}^{p}\left(1-\gamma_{j})I\{\gamma_{j}>k\right\}}{\sum_{j=1}^{p}I\{\gamma_{j}>k\}}<\alpha \right\}$$

**Table 4.** Gene names and selection proportions for ovarian cancer dataset

PI3	PPP3CA	CCR7	SDF2L1	D4S234E	VSIG4	DAP	IL7R
0.7	0.379	0.293	0.286	0.229	0.171	0.136	0.136
TBP	ACSL3	SLAMF7	UBD	IL2RG	GALNT10	FLJ20323	RNF128
0.121	0.114	0.1	0.1	0.064	0.057	0.05	0.05

Notably, $k^{*}$ is computed for each fit and is a threshold used to control the Bayesian FDR at significance level *α*, which we have set as α=0.10 (Newton *et al.*, 2004). Promisingly, the most frequently selected gene, *PI3*, has a known, albeit limited, role in ovarian cancer, a disease characterized by copy number aberration. Clauss *et al.* (2010) reported the first link of *PI3* gene product (elafin) to ovarian cancer (Clauss *et al.*, 2010). Elafin, a serine proteinase inhibitor involved in inflammation and wound healing, is overexpressed in ovarian cancer and overexpression is associated with poor overall survival and is due, in part, to genomic gains on chromosome 20q13.12, a locus frequently amplified in ovarian carcinomas. There is less known about the gene encoding the alpha isoform of the calcineurin catalytic subunit (*PPP3CA*) in ovarian cancer. A recent report indicates that higher expression of calcineurin predicts poor prognosis in ovarian cancer, particularly those of serous histology (Xin *et al.*, 2019). It is also plausible that other know functions of calcineurin/nuclear factor of activated T cells, in controlling adaptive T-cell function or innate immunity (Fric *et al.*, 2012), in this cancer that warrants further investigation. Finally, *CCR7*, the third most abundant gene was recently reported, in single cell RNA-seq analysis, to be emphasized in high-grade serous ovarian cancer (Izar *et al.*, 2020).

### 4.2 Breast cancer dataset

The second dataset we analyzed is a transcriptomic dataset collected from patients with breast cancer, where the outcome of interest is overall survival (Yau *et al.*, 2010). The dataset consists of *n *=* *682 samples and *p *=* *9168 features which we preprocessed as before, leaving *p *=* *4, 584 features. Within the dataset 454 observations are right censored, corresponding to a censoring rate of 66.5%.

As in the previous section, we set $a_{0}=p/100$ and $b_{0}=p$, and tuned the prior parameter *λ* via 10-fold cross validation using the same set Λ. Supplementary Table S3 reports the ELBO, ELL and c-index averaged across the validation and training sets. We note that the model is not particularly sensitive to the value of *λ*. Furthermore, an assessment of the convergence diagnostics for λ=2.5, presented in Supplementary Figure S12, carries a similar interpretation as with the TCGA data.

Table 5 reports the names and selection proportions of the genes within the dataset. The most frequently selected gene, Rho GTPase activating protein 28 (*ARHGAP28*) is a negative regulator of RhoA. There is paucity of data on this gene in cancer generally, however, a report by Planche *et al.* (2011) identified the gene as downregulated in reactive stroma of prostate tumors. Further assessment of this gene in breast cancer is warranted. Notably, *NEK2*, *GREM1* and *ABCC5* have been examined in the biomedical literature and have been associated with cancer cell proliferation and metastasis. More specially, overexpression of *NEK2* induces epithelial-to-mesenchymal transition, a process which leads to functional changes in cell invasion, overexpression of *GREM1* has been associated with metastasis and poor prognosis, and *ABCC5* has been associated with breast cancer skeletal metastasis (Mourskaia *et al.*, 2012; Park *et al.*, 2020; Rivera-Rivera *et al.*, 2021). As with the TCGA data, it is encouraging that genes with pre-existing biological interpretation have been selected by our model.

**Table 5.** Gene names and selection proportions for the breast cancer dataset

ARHGAP28	NEK2	ABCC5	GREM1	DUSP4	ITGA5	CCL2	IGFBP7
0.386	0.25	0.2	0.2	0.193	0.193	0.164	0.143
NFE2L3	TRPC1	PKMYT1	DDX31	EMILIN1	SSPN	ABO	HSPC072
0.114	0.114	0.1	0.086	0.086	0.086	0.079	0.079

Finally, we want to highlight that our method, in contrast to the methods compared in Section 3.2.2, quantifies the uncertainty of *β*. Crucially, the availability of uncertainty serves as a powerful inferential tool for computing (variational) posterior probabilities with respect to risk scores ($\beta^{⊤}x)$. Such probabilities can be useful in comparing patients between one another, or assessing the risk of patients against chosen benchmarks (depending on the aims of the practitioner).

**Fig. 2.**
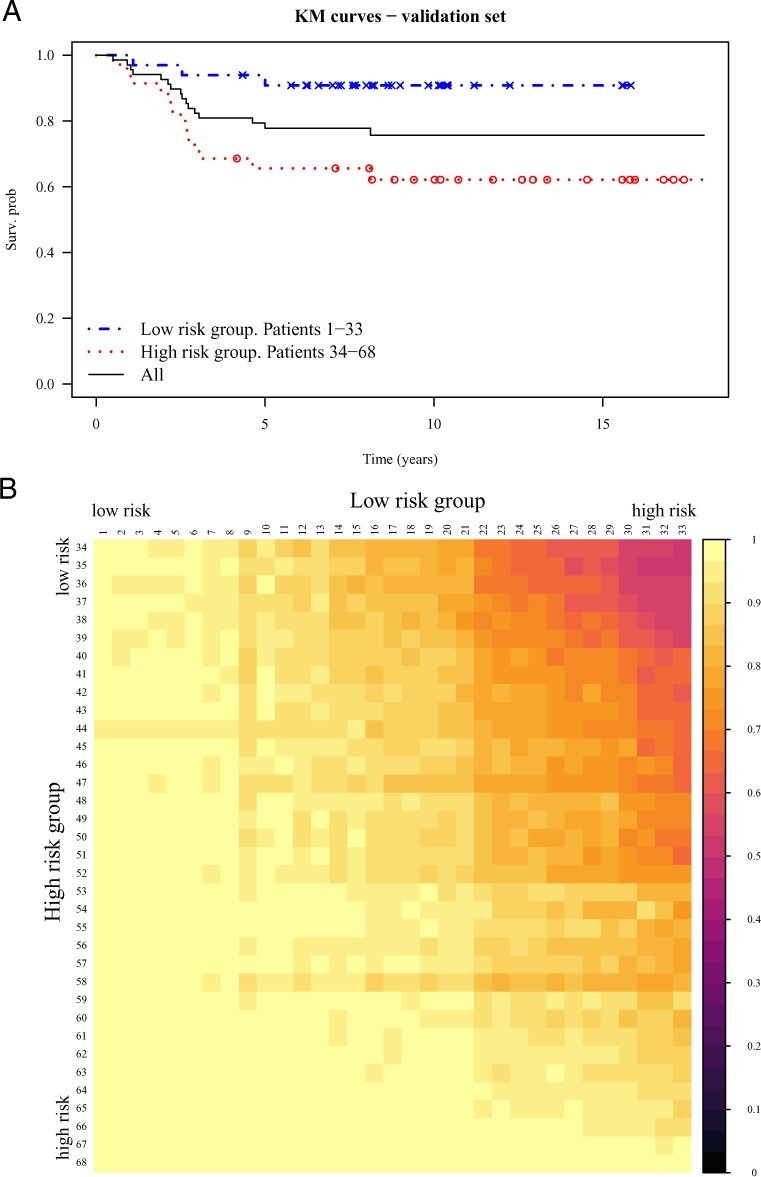
(**A**) Kaplan–Meier curves for patients in low- and high-risk groups. (**B**) Comparison of patients in the low- and high-risk groups (ordered by $\hat{\eta }$)—within each cell the (variational) posterior probability patient in row *i* is at greater risk than patient in column *j* is computed. Samples are taken from the second validation fold and the fit with λ=2.5 is used

To demonstrate, we opt to compute the posterior probability that one patient is at greater risk than another, formally, $\tilde{\Pi }(\beta^{⊤}x_{i}\geq \beta^{⊤}x_{j})$, where i≠j. To illustrate the application, we split patients into low- and high-risk groups based on the estimated prognostic index, ${\hat{\eta }}_{i}={\hat{\beta }}^{⊤}x_{i}$, where $\hat{\beta }$ is the posterior mean. Patients with prognostic index less than the median (computed for the training set) are considered low risk, whilst patients with prognostic index greater than or equal to the median are considered high risk. The Kaplan–Meier curves for these groups are shown in Figure 2A. Critically, Bayesian approaches that only compute the MAP of *β* are only able to provide a point estimates for $\hat{\eta }$. In turn, our method is able to provide uncertainty around this quantity and therefore with respect to the ranking of the patients. For instance, in Figure 2B, we present the posterior probabilities comparing the risks between patients. We observe that the highest risk patients in the low-risk group are comparable to the lowest risk patients in the high-risk group, and that the highest risk patients within the high-risk group are with high probability more at risk than the patients within the low-risk group.

## 5 Discussion

Variable selection and effect estimation for high-dimensional survival data has been an issue of great interest in recent years, particularly given the ever growing production of large scale omics data. However, the high-dimensionality and heterogeneity in the predictors, alongside the censoring in the response, makes the analysis a non-trivial task. While many recent methods have tackled these issues through a Bayesian approach, due to long compute times they often only produce point estimates rather than the full posterior and thereby fall short in providing the full Bayesian machinery.

We have bridged this gap and developed a scalable and interpretable mean-field variational approximation for Bayesian PHMs with a spike-and-slab prior. We have demonstrated that the resulting variational posterior displays similar performance to the posterior obtained via MCMC whilst requiring a fraction of the compute time. Furthermore, we have demonstrated via an extensive simulation study that our proposed method performs comparably to state-of-the art Bayesian variable selection methods.

Finally, we have demonstrated that our method can be used for variable selection on two real world transcriptomics datasets, giving rise to results with pre-existing biological interpretations, thereby validating the practical utility of our method. We have also shown that the risk of patients can be compared through (variational) posterior probabilities, highlighting that the availability of a posterior distribution can be a powerful inferential tool. For illustrative purposes, we examined the pairwise probabilities of patients grouped based on the prognostic index, however, patients could have alternatively been compared to other baselines, e.g. the feature vector corresponding to median prognostic index. Furthermore, although this is not an aspect we have considered, grouping based on: age, cancer status, stage etc. may yield insightful results for practitioners and bioinformaticians.

A natural extension of our work would be to develop approximations with relaxed independence assumptions by using a more flexible variational family (Ning, 2021). Finally, we would like to highlight that improving the uncertainty quantification is an active area of research in the general VI community, see e.g. (Jerfel *et al.*, 2021).

## Supplementary Material

btac416_Supplementary_Data
