# Supplementary material for: Variational Bayes for high-dimensional proportional hazards models with applications within gene expression
Source: Bioinformatics. 2022 Jun 25;38(16):3918–26. doi: 10.1093/bioinformatics/btac416 (PMC9364383; doi:10.1093/bioinformatics/btac416)
Supplement: btac416_Supplementary_Data [file btac416_supplementary_data.pdf]

A Variational algorithm

Recall that our mean-field variational family is given by

$$\mathcal{Q} = \left\{ Q_{\mu,\sigma,\gamma} = \bigotimes_{j=1}^{p} \left[\gamma_j N(\mu_j, \sigma_j^2) + (1-\gamma_j)\delta_0 \right] : \mu_j \in \mathbb{R}, \sigma_j \in \mathbb{R}^+, \gamma_j \in [0,1] \right\}, \tag{1}$$

and the posterior is given by

$$d\Pi(\beta|\mathcal{D}) = \Pi_D^{-1} e^{l_p(\mathcal{D};\beta)} d\Pi(\beta), \tag{2}$$

where $l_p = \log L_p$ is the log (partial) likelihood and Π_D is the normalising constant. Our aim is to evaluate the KL divergence between an element $Q_{\mu,\sigma,\gamma}$ of the variational family and the posterior $\Pi(\cdot|\mathcal{D})$ as a function of μ_j, σ_j and γ_j, whilst keeping all other parameters fixed. Due to the discrete components of the prior (4), one has to be careful with the different terms since they may not be absolutely continuous with respect to one another (as measures), and hence may not have densities.

A.1 Update equations for μ_j and σ_j

We first compute the KL divergence between $Q_{\mu,\sigma,\gamma}$ and the posterior $\Pi(\cdot|\mathcal{D})$, conditional on $z_j = 1$, as a function of μ_j and σ_j. We recall the notation μ_{-j}, which refers to all the components of $(\mu_1, \ldots, \mu_p)$ except μ_j. Firstly, since both the prior (4) and variational family (6) consist of factorisable distributions, the Radon-Nikodym derivative between them also factorizes

$$\frac{dQ_{\mu,\sigma,\gamma}}{d\Pi}(\beta) = \prod_{j=1}^{n} \frac{dQ_j}{d\Pi_j}(\beta_j), \tag{3}$$

where $Q_j = \gamma_j N(\mu_j, \sigma_j^2) + (1-\gamma_j)\delta_0$ and $\Pi_j = \bar{w}\text{Lap}(\lambda) + (1-\bar{w})\delta_0$ with $\bar{w} = a_0/(a_0+b_0)$. The latter expression for the prior follows from integrating out the hierarchical representation (4), whereupon we have weights equal to the prior mean weight $\bar{w}$.

Since the variational probability distribution of β_j conditional on $z_j = 1$ (i.e. the slab component) is singular with respect to the Dirac measure δ_0, in this case it is sufficient to consider only the continuous part of the prior measure in the denominator of the Radon-Nikodym derivative, that is

$$\frac{dQ_{\mu_j,\sigma_j|z_j=1}}{d\Pi_j}(\beta_j) = \frac{dN(\mu_j, \sigma_j^2)}{\bar{w} d\text{Lap}(\lambda)}(\beta_j).$$

Using the above facts, $\mathrm{KL}(Q_{\mu,\sigma,\gamma|z_j=1} \parallel \Pi(\cdot|\mathcal{D}))$ equals, as a function of μ_j and σ_j,

$$\begin{aligned}
\mathbb{E}_{\mu,\sigma,\gamma|z_j=1}\left[\log \frac{dQ}{d\Pi(\cdot|\mathcal{D})}\right] &= \mathbb{E}_{\mu,\sigma,\gamma|z_j=1}\left[\log \frac{dQ_{\mu,\sigma,\gamma}}{d\Pi} - l_p(\mathcal{D};\beta) + \log \Pi_D\right] \\
&= \mathbb{E}_{\mu,\sigma,\gamma|z_j=1}\left[\log\left(\frac{dN(\mu_j,\sigma_j^2)}{\bar{w}d\mathrm{Lap}(\lambda)}(\beta_j)\prod_{k\neq j}\frac{dQ_k}{d\Pi_k}(\beta_k)\right) - l_p(\mathcal{D};\beta) + \log \Pi_D\right] \\
&= \mathbb{E}_{\mu,\sigma,\gamma|z_j=1}\left[\log\left(\prod_{k\neq j}\frac{dQ_k}{d\Pi_k}(\beta_k)\right) + \log\left(\frac{1}{\sqrt{2\pi\sigma_j^2}}e^{-\frac{(\beta_j-\mu_j)^2}{2\sigma_j^2}}\frac{2}{\lambda\bar{w}}e^{\lambda|\beta_j|}\right) - l_p(\mathcal{D};\beta) + \log \Pi_D\right] \\
&= \mathbb{E}_{\mu,\sigma,\gamma|z_j=1}\left[\lambda|\beta_j| - \log\sigma_j - \frac{(\beta_j-\mu_j)^2}{2\sigma_j^2} - l_p(\mathcal{D};\beta)\right] + C \\
&= \mathbb{E}_{\mu,\sigma,\gamma|z_j=1}\left[\lambda|\beta_j| - \log\sigma_j - \frac{(\beta_j-\mu_j)^2}{2\sigma_j^2} - \sum_{\{i:\delta_i=1\}}\left(\beta^\top x_i - \log\sum_{r\in R(t_i)}\exp(\beta^\top x_r)\right)\right] + C,
\end{aligned}$$

where the constant C is independent of μ_j and σ_j and may vary from line to line. Since $|\beta_j|$ has a folded normal distribution, it has expectation $\sigma_j\sqrt{2/\pi}e^{-\mu_j^2/(2\sigma_j^2)} + \mu_j(1-2\Phi(-\mu_j/\sigma_j))$, where Φ is the CDF of the standard normal distribution. The previous display equals

$$\begin{aligned}
&\sum_{\{i:\delta_i=1\}}\left(\mathbb{E}_{\mu,\sigma,\gamma|z_j=1}\left[\log\sum_{r\in R(t_i)}\exp(\beta^\top x_r)\right] - \mu_j x_{ij}\right) \\
&\quad + \lambda\sigma_j\sqrt{2/\pi}e^{-\mu_j^2/(2\sigma_j^2)} + \lambda\mu_j(1-2\Phi(-\mu_j/\sigma_j)) - \log\sigma_j + C \\
\leq &\sum_{\{i:\delta_i=1\}}\left(\log\sum_{r\in R(t_i)}\mathbb{E}_{\mu,\sigma,\gamma|z_j=1}\left[\exp(\beta^\top x_r)\right] - \mu_j x_{ij}\right) \\
&\quad + \lambda\sigma_j\sqrt{2/\pi}e^{-\mu_j^2/(2\sigma_j^2)} + \lambda\mu_j(1-2\Phi(-\mu_j/\sigma_j)) - \log\sigma_j + C \\
= &\sum_{\{i:\delta_i=1\}}\left(\log\sum_{r\in R(t_i)}M(x_{rj},\mu_j,\sigma_j)P_j(x_r,\mu,\sigma,\gamma) - \mu_j x_{ij}\right) \\
&\quad + \lambda\sigma_j\sqrt{2/\pi}e^{-\mu_j^2/(2\sigma_j^2)} + \lambda\mu_j(1-2\Phi(-\mu_j/\sigma_j)) - \log\sigma_j + C, \qquad (4)
\end{aligned}$$

where $M(x_{rj},\mu_j,\sigma_j) = \exp(\mu_j x_{rj} + \frac{1}{2}\sigma_j^2 x_{rj}^2)$ and $P_j(x_r,\mu,\sigma,\gamma) = \prod_{k\neq j}(\gamma_k M(x_{rk},\mu_k,\sigma_k) + (1-\gamma_k))$ and we have used Jensen's inequality. Noting, to evaluate an expression for $\mathbb{E}_{\mu,\sigma,\gamma|z_j=1}\left[e^{\beta^\top x_r}\right]$ we exploit the independence structure of β, i.e. $\beta_j \overset{\text{ind}}{\sim} \gamma_j N(\mu_j,\sigma^2) + (1-\gamma_j)\delta_0$, and hence $\mathbb{E}_{\mu,\sigma,\gamma|z_j=1}\left[e^{\beta^\top x_r}\right] = \mathbb{E}_{\mu_j,\sigma_j,\gamma_j|z_j=1}\left[e^{\beta_j x_{rj}}\right]\prod_{k\neq j}\mathbb{E}_{\mu_k,\sigma_k,\gamma_k}\left[e^{\beta_k x_{rk}}\right]$.

The last display thus provides an upper bound for the KL divergence and hence a surrogate objective function. Minimising this expression with respect to either μ_j or σ_j gives the same minimisers as minimising the objective functions (8) or (9), respectively, and hence gives our update equations.

A.2 Update equation for γ_j

In a similar way, the KL divergence between $Q_{\mu,\sigma,\gamma}$ and $\Pi(\cdot|\mathcal{D})$ as of function of γ_j equals

$$\mathbb{E}_{\mu,\sigma,\gamma}\left[\log\left(\prod_{k\neq j}\frac{dQ_k}{d\Pi_k}(\beta_k)\right)+\log\frac{d[\gamma_j N(\mu_j,\sigma_j^2)+(1-\gamma_j)\delta_0]}{d[\bar{w}\mathrm{Lap}(\lambda)+(1-\bar{w})\delta_0]}(\beta_j)-l_p(\mathcal{D};\beta)\right]+C.$$

We now split this expression in terms of the events $z_j=1$ or 0. Noting that with $Q_{\mu,\sigma,\gamma}$-probability one, $\beta_j=0$ if and only if $z_j=0$, the last display can rewritten as

$$\begin{aligned}
&\mathbb{E}_{\mu,\sigma,\gamma}\left[\mathbb{I}_{\{z_j=1\}}\log\frac{\gamma_j dN(\mu_j,\sigma_j^2)}{\bar{w}d\mathrm{Lap}(\lambda)}(\beta_j)+\mathbb{I}_{\{z_j=0\}}\log\frac{(1-\gamma_j)}{(1-\bar{w})}-l_p(\mathcal{D};\beta)\right]+C\\
&\quad=\mathbb{E}_{\mu,\sigma,\gamma}\left[\mathbb{I}_{\{z_j=1\}}\left(\log\left(\frac{\gamma_j}{\bar{w}}\frac{\sqrt{2}}{\sqrt{\pi}\sigma_j\lambda}\right)+\lambda|\beta_j|-\frac{(\beta_j-\mu_j)^2}{2\sigma_j^2}\right)+\mathbb{I}_{\{z_j=0\}}\log\frac{(1-\gamma_j)}{(1-\bar{w})}-l_p(\mathcal{D};\beta)\right]+C\\
&\quad=\gamma_j\left\{\log\frac{\gamma_j}{\bar{w}}+\log\frac{\sqrt{2}}{\sqrt{\pi}\sigma_j\lambda}+\lambda\sigma_j\sqrt{2/\pi}e^{-\mu_j^2/(2\sigma_j^2)}+\lambda\mu_j(1-2\Phi(-\mu_j/\sigma_j))-\frac{1}{2}\right\}\\
&\qquad+(1-\gamma_j)\log\frac{(1-\gamma_j)}{(1-\bar{w})}+\sum_{\{i:\delta_i=1\}}\left(\mathbb{E}_{\mu,\sigma,\gamma}\left[\log\sum_{r\in R(t_i)}\exp(\beta^\top x_r)\right]-\gamma_j\mu_j x_{ij}\right)+C,
\end{aligned}$$

where C does not depend on γ_j. Upper bounding the expectation in the last display using Jensen's inequality,

$$\begin{aligned}
&\mathbb{E}_{\mu,\sigma,\gamma}\left[\log\sum_{r\in R(t_i)}\exp(\beta^\top x_r)\right]\\
&\quad=\sum_{\{i:\delta_i=1\}}\left(\gamma_j\mathbb{E}_{N(\mu_j,\sigma_j^2)\otimes Q_{-j}}\left[\log\sum_{r\in R(t_i)}e^{\beta^\top x_r}\right]+(1-\gamma_j)\mathbb{E}_{\delta_0\otimes Q_{-j}}\left[\log\sum_{r\in R(t_i)}e^{\beta^\top x_r}\right]\right)\\
&\quad\leq\sum_{\{i:\delta_i=1\}}\left(\gamma_j\log\sum_{r\in R(t_i)}\mathbb{E}_{N(\mu_j,\sigma_j^2)\otimes Q_{-j}}\left[e^{\beta^\top x_r}\right]+(1-\gamma_j)\log\sum_{r\in R(t_i)}\mathbb{E}_{\delta_0\otimes Q_{-j}}\left[e^{\beta^\top x_r}\right]\right)
\end{aligned}$$

$$= \sum_{\{i:\delta_i=1\}} \left(\gamma_j \log \sum_{r \in R(t_i)} M(x_{rj}, \mu_j, \sigma_j) P_j(x_r, \mu, \sigma, \gamma) + (1-\gamma_j) \log \sum_{r \in R(t_i)} P_j(x_r, \mu, \sigma, \gamma) \right).$$

Substituting this into the second to last display, we can upper bound the KL divergence as a function of γ_j by

$$\gamma_j \sum_{\{i:\delta_i=1\}} \left(\log \sum_{r \in R(t_i)} M(x_{rj}, \mu_j, \sigma_j) P_j(x_r, \mu, \sigma, \gamma) - \log \sum_{r \in R(t_i)} P_j(x_r, \mu, \sigma, \gamma) - \mu_j x_{ij} \right) +$$
$$\gamma_j \left(\lambda \sigma_j \sqrt{2/\pi} e^{-\mu_j^2/(2\sigma_j^2)} + \lambda \mu_j (1 - 2\Phi(-\mu_j/\sigma_j)) + \log \frac{\sqrt{2}}{\sqrt{\pi}\sigma_j \lambda} - \frac{1}{2} + \log \frac{\gamma_j}{1-\gamma_j} - \log \frac{a_0}{b_0} \right) \quad (5)$$
$$+ \log(1-\gamma_j) + C$$

where C does not depend on γ_j and we have used $\bar{w} = a_0/(a_0 + b_0)$. Setting the derivative with respect to γ_j of the last equation equal to zero and rearranging gives the update equation (10) for γ_j.

B Goodness of fit measures

A commonly used measure of goodness of fit in the VI literature is the evidence lower bound (ELBO), which acts as a lower bound for the Bayesian marginal likelihood and is defined as

$$\mathcal{L}_Q = \mathbb{E}_Q \left[\log L_p \right] - \mathrm{KL}(Q \| \Pi), \quad (6)$$

with $Q \in \mathcal{Q}$ [Bishop, 2006]. Intuitively, the ELBO trades-off how well the model fits the data (first term) with how close it is to the prior (second term). Picking the parameter that maximizes the ELBO is the variational analogue of empirical Bayes, which selects parameters by maximising the marginal likelihood. Since VI is used exactly when the marginal likelihood is intractable, it is natural to use the ELBO, which measures the quality of the variational approximation [Bishop, 2006].

Similar to our derivation of the coordinate-ascent update expression for γ_j, we exploit the

product structure of our variational distribution to evaluate an expression for the ELBO.

$$
\begin{aligned}
\mathcal{L}_Q =& -\mathbb{E}_{\mu,\sigma,\gamma}\left[\log \frac{dQ_{\mu,\sigma,\gamma}}{d\Pi}(\beta) - l_p(\mathcal{D};\beta)\right] \\
=& -\mathbb{E}_{\mu,\sigma,\gamma}\left[\sum_{j=1}^{p}\left(\mathbb{I}_{\{z_j=1\}}\log \frac{\gamma_j dN(\mu_j,\sigma_j^2)}{\bar{w}_j d\mathrm{Lap}(\lambda)}(\beta_j) + \mathbb{I}_{\{z_j=0\}}\log\frac{(1-\gamma_j)}{(1-\bar{w}_j)}\right) - l_p(\mathcal{D};\beta)\right] \\
=& -\sum_{j=1}^{p}\left(\mathbb{E}_{Q_j}\left[\mathbb{I}_{\{z_j=1\}}\log \frac{\gamma_j dN(\mu_j,\sigma_j^2)}{\bar{w} d\mathrm{Lap}(\lambda)}(\beta_j) + \mathbb{I}_{\{z_j=0\}}\log\frac{(1-\gamma_j)}{(1-\bar{w})}\right]\right) + \mathbb{E}_{\mu,\sigma,\gamma}\left[l_p(\mathcal{D};\beta)\right] \\
=& -\sum_{j=1}^{p}\left(\gamma_j\left\{\lambda\sigma_j\sqrt{2/\pi}e^{-\mu_j^2/(2\sigma_j^2)} + \lambda\mu_j(1-2\Phi(-\mu_j/\sigma_j)) + \log\frac{\sqrt{2}}{\sqrt{\pi}\sigma_j\lambda} - \frac{1}{2}\right.\right. \\
&\left.\left. + \log\frac{\gamma_j}{1-\gamma_j} - \log\frac{a_0}{b_0}\right\} + \log(1-\gamma_j) - \log(1-\bar{w})\right) + \mathbb{E}_{\mu,\sigma,\gamma}\left[l_p(\mathcal{D};\beta)\right] \qquad (7)
\end{aligned}
$$

As we cannot evaluate a closed-form expression for $\mathbb{E}_{\mu,\sigma,\gamma}\left[l_p(\mathcal{D};\beta)\right]$, we use Monte Carlo integration to estimate this quantity, i.e. we compute

$$
\widehat{\mathcal{L}}_Q = \frac{1}{B}\sum_{i=1}^{B}\log L_p(\mathcal{D};\beta^{(i)}) - \mathrm{KL}(Q\|\Pi), \quad Q \in \mathcal{Q}, \qquad (8)
$$

where B are the number of Monte Carlo samples and $\beta^{(i)} \overset{\text{iid}}{\sim} Q$ for $i = 1, \dots, B$. Notably, the elements of $\beta^{(i)} = (\beta_1^{(i)}, \dots, \beta_p^{(i)})^\top \in \mathbb{R}^p$ are given by sampling $\beta_j^{(i)} \overset{\text{ind}}{\sim} N(\mu_j, \sigma_j^2)$ with probability γ_j or taking $\beta_j^{(i)} = 0$ with probability $1 - \gamma_j$ for $j = 1, \dots, p$. The remaining term can be evaluated explicitly as

$$
\begin{aligned}
\mathrm{KL}(Q\|\Pi) = & \sum_{j=1}^{p}\left(\gamma_j\left\{\lambda\sigma_j\sqrt{\frac{2}{\pi}}e^{-\frac{\mu_j^2}{2\sigma_j^2}} + \log\frac{\sqrt{2}}{\sqrt{\pi}\sigma_j\lambda} - \frac{1}{2}\right.\right. \qquad (9) \\
& \left.\left. + \lambda\mu_j(1-2\Phi(-\frac{\mu_j}{\sigma_j})) + \log\frac{\gamma_j}{1-\gamma_j} - \log\frac{a_0}{b_0}\right\} + \log(1-\gamma_j)\right).
\end{aligned}
$$

Although popular, model selection based on the ELBO is not justified theoretically and can be sensitive to the setting; therefore we consider additional goodness of fit measures. One such measure, proposed by Nott et al. (2012), involves using the variational posterior to approximate

the log-predictive density score (LPDS) on an held out testing dataset, defined as

$$\text{LPDS} = \log \int L_p\left(\mathcal{D}_{\text{test}}; \beta\right) d\tilde{\Pi}(\beta|\mathcal{D}_{\text{train}}) = \log \mathbb{E}_{\tilde{\Pi}}\left[L_p\left(\mathcal{D}_{\text{test}}; \beta\right)\right],$$

where $\mathcal{D}_{\text{test}}$ is the held out test set and $\mathcal{D}_{\text{train}}$ is the training set used to compute $\tilde{\Pi}$ [Nott et al., 2012]. By Jensen's inequality,

$$\log \mathbb{E}_{\tilde{\Pi}}\left[L_p\left(\mathcal{D}_{\text{test}}|\beta\right)\right] \leq \mathbb{E}_{\tilde{\Pi}}\left[\log L_p\left(\mathcal{D}_{\text{test}}|\beta\right)\right]$$

Hence, given that computing the ELBO for a validation set involves computing $\mathbb{E}_{\tilde{\Pi}}\left[\log L_p\left(\mathcal{D}_{\text{test}}; \beta\right)\right]$, we can cheaply obtain an upper bound for the LPDS to use as a goodness of fit measure.

A further goodness of fit measure specific to survival models is the concordance index (c-index), defined as

$$k = \mathbb{P}(\boldsymbol{T}_i > \boldsymbol{T}_j | \eta_j > \eta_i), \quad i \neq j,$$

where $\eta_k = \beta_0^\top x_k$ is referred to as the *prognostic index*. Intuitively, for two observations $(t_i, \delta_i = 1, x_i)$ and $(t_j, \delta_j = 1, x_j)$, when $\eta_j > \eta_i$ we would expect $t_i > t_j$. This remark follows from the form of the hazard function under the PHM, where we assume $h(t) = h_0(t)\exp(\beta^\top x) = h_0(t)\exp(\eta)$. Therefore, when $\eta_j > \eta_i$ the hazard rate $h(t_j) > h(t_i)$ and thus we would expect t_j to have failed before t_i. Often the c-index is estimated by,

$$\widehat{k} = \frac{\sum_{i=1}^n \sum_{j>i} \mathbb{I}(t_i < t_j)\mathbb{I}(\widehat{\eta}_i > \widehat{\eta}_j)\delta_i + \mathbb{I}(t_j < t_i)\mathbb{I}(\widehat{\eta}_j > \widehat{\eta}_i)\delta_j}{\sum_{i=1}^n \sum_{j>i} \mathbb{I}(t_i < t_j)\delta_i + \mathbb{I}(t_j < t_i)\delta_j},$$

where the prognostic index is estimated using $\widehat{\eta}_j = \widehat{\beta}^\top x_j$ for a given point estimate $\widehat{\beta}$ of β, and $\mathbb{I}(\cdot)$ is the indicator function [Harrell et al., 1982]. Notably, the c-index is not robust to censoring and tends to overestimate k when there is a high degree of censoring [Gonen and Heller, 2005].

C Simulation study

C.1 Markov chain Monte Carlo sampler

To construct our sampler we note that the model given by (5) can be reformulated such that the prior is given by:

$$\begin{aligned} \beta_j &\overset{\text{iid}}{\sim} \text{Laplace}(\lambda) \\ z_j|w_j &\overset{\text{ind}}{\sim} \text{Bernoulli}(w_j) \\ w_j &\overset{\text{iid}}{\sim} \text{Beta}(a_0, b_0) \end{aligned} \tag{10}$$

and likelihood as $p(\mathcal{D}|\beta, z) = L_p(\mathcal{D}; \beta \circ z)$ where $\circ$ denotes the element wise product, i.e. $(\beta \circ z)_j = \beta_j z_j$.

Algorithm 1 details our Gibbs sampler for the posterior in (5) based on the above formulation. Notably, we introduce the notation $x_{k:l} := (x_i)_{i=k}^{l}$, for $1 \leq l < k \leq p,\ x \in \mathbb{R}^p$, and denote $\beta^{(i)} \in \mathbb{R}^p$, $z^{(i)} \in \{0, 1\}^p$ and $w^{(i)} \in [0, 1]^p$ as the MCMC samples.

Algorithm 1 Spike and Slab MCMC sampler

Require: K, the Metropolis-Hastings proposal kernel, N: number of samples.
Initialise $z^{(0)}, \beta^{(0)}, w^{(0)}$
for $i = 1, \dots N$ **do**
  for $j = 1, \dots p$
    $w_j^{(i)} \sim w_j|\mathcal{D}, \beta_{1:p}^{(i-1)}, z_{1:p}^{(i-1)}, w_{1:j-1}^{(i-1)}, w_{j+1:p}^{(i-1)}$
  for $j = 1, \dots p$
    $z_j^{(i)} \sim z_j|\mathcal{D}, \beta_{1:p}^{(i-1)}, z_{1:(j-1)}^{(i)}, z_{(j+1):p}^{(i-1)}, w_{1:p}^{(i)}$
  for $j = 1, \dots p$
    $\beta_j^{(i)} \sim \beta_j|\mathcal{D}, \beta_{1:(j-1)}^{(i-1)}, \beta_{(j+1):p}^{(i-1)}, z_{1:p}^{(i)}, w_{1:p}^{(i)}$

Ignoring the superscript for clarity, the distribution $w_j|\mathcal{D}, \beta, z, w_{-j}$ is conditionally independent of $\mathcal{D}, \beta, z$ and w_{-j}. Therefore, $w_j^{(i)}$ is sampled iid. from the prior of w_j, i.e. $w_j^{(i)} \overset{\text{iid.}}{\sim} \text{Beta}(a_0, b_0)$. Regarding $z_j^{(i)}$, the conditional density

$$\begin{aligned} p(z_j|\mathcal{D}, \beta, z_{-j}, w) &\propto p(\mathcal{D}|\beta, z_{-j}, z_j, w)\pi(z_j|\beta, z_{-j}, w). \\ &= p(\mathcal{D}; \beta, z)\pi(z_j|w_j). \end{aligned} \tag{11}$$

As z_j is discrete, evaluating the RHS of (11) for $z_j = 0$ and $z_j = 1$, gives the unnormalised conditional probabilities. Summing gives the normalisation constant and thus we can sample z_j from a Bernoulli distribution with parameter

$$p = \frac{p(z_j = 1|\mathcal{D}, \beta, z_{-j}, w)}{p(z_j = 0|\mathcal{D}, \beta, z_{-j}, w) + p(z_j = 1|\mathcal{D}, \beta, z_{-j}, w)}. \tag{12}$$

Finally, to sample from $\beta_j^{(i)}$ we use a Metropolis-Hastings within Gibbs step, wherein a proposal $\beta_j^{(i)}$ is sampled from a random-walk proposition kernel K. The proposal is then accepted with probability A or rejected with probability $1 - A$, in which case $\beta_j^{(i)} \leftarrow \beta_j^{(i-1)}$. Noting A is given by,

$$A = \min\left(1, \frac{p(\mathcal{D}; \beta_{-j}, \beta_j^{(i)}, z^{(i)})\pi(\beta_j^{(i)})}{p(\mathcal{D}; \beta_{-j}, \beta_j^{(i-1)}, z^{(i)})\pi(\beta_j^{(i-1)})} \frac{K(\beta_j^{(i-1)}|\beta_j^{(i)})}{K(\beta_j^{(i)}|\beta_j^{(i-1)})}\right) \tag{13}$$

Within our implementation we let $K = N\left(\beta_j^{(i-1)}, \sigma_k^2 \sigma_s^{2(1-z_j^{(i-1)})}\right)$ with $\sigma_k = 0.2$ and $\sigma_s = 10$. The implementation of the sampler is available as an `R` package that can be installed from `https://github.com/mkomod/survival-ss`.

D Sensitivity analysis

D.1 Sensitivity to starting values

To examine the sensitivity with respect to the initialization of μ, σ and γ we generate data as describe in Section 3.1 taking $(n, p, s, c) = (200, 1000, 10, 0.25)$.

D.1.1 Sensitivity to μ

To examine the sensitivity with respect to initialization of μ we compared four different methods:

- Random: where $\mu_j \overset{\text{iid.}}{\sim} N(0, 1)$ for $j = 1, \dots, p$.
- Ridge: where μ is the MLE under the ridge penalty.
- Elastic Net: where μ is the MLE under the elastic net penalty (equal mixture of ridge and LASSO penalties).
- LASSO: where μ is the MLE under the LASSO penalty.

In the last three cases a regularization hyperparameter of $100\lambda_{\min}$ is used, where $\lambda_{\min}$ is the hyperparameter wherein all estimates coefficients are equal to zero. In order to compute the MLE under the different penalties we use `glmnet`. To evaluate the different initialization methods we compute the ℓ_2-error, ℓ_1-error, TPR, FDR, AUC and runtime, presenting the median, lower (5%) and upper (95%) quantiles across 100 runs in Table 1.

Examining Table 1, we notice that within setting 1 the initialization method does not impact the performance of the method with all methods performing equally. It is worth noting the runtime for random initialization is largest, meaning it takes the algorithm longer to converge given a poor initialization. Within settings 2-4, the initialization methods can have a substantial effect on performance. For instance, within settings 3 initialization with ridge yields a median ℓ_2-error of 1.019, whereas initialization with the LASSO penalty gives a median ℓ_2-error of 0.394. Furthermore, poor initialization has an effect on the variable selection of the method (Table 1). Overall, initialization of μ using the LASSO gave the best models, obtaining best metrics across the different settings. In some cases however, initialization with the LASSO can be slower than other methods e.g. the elastic net, which produced comparable models.

Setting	Init. Meth.	ℓ_2-error	ℓ_1-error	TPR	FDR	AUC	Runtime
Setting 1	Random	0.368 (0.21, 0.70)	0.999 (0.52, 1.86)	1.000 (0.90, 1.00)	0.000 (0.00, 0.00)	1.000 (1.00, 1.00)	19.3s (15.6s,31.3s)
	Ridge	0.368 (0.21, 0.70)	0.999 (0.52, 1.86)	1.000 (0.90, 1.00)	0.000 (0.00, 0.00)	1.000 (1.00, 1.00)	7.1s (4.8s,13.0s)
	Elas. Net.	0.368 (0.21, 0.70)	0.999 (0.52, 1.86)	1.000 (0.90, 1.00)	0.000 (0.00, 0.00)	1.000 (1.00, 1.00)	7.3s (4.5s,12.3s)
	LASSO	0.368 (0.21, 0.70)	1.000 (0.52, 1.86)	1.000 (0.90, 1.00)	0.000 (0.00, 0.00)	1.000 (1.00, 1.00)	13.8s (9.4s,19.8s)
Setting 2	Random	0.848 (0.26, 3.06)	1.988 (0.68, 10.34)	0.900 (0.20, 1.00)	0.000 (0.00, 0.50)	1.000 (0.75, 1.00)	29.3s (19.0s,44.9s)
	Ridge	0.838 (0.26, 3.43)	1.970 (0.69, 11.70)	0.900 (0.30, 1.00)	0.000 (0.00, 0.50)	1.000 (0.74, 1.00)	6.9s (3.0s,12.3s)
	Elas. Net.	0.565 (0.24, 1.72)	1.566 (0.67, 4.58)	1.000 (0.70, 1.00)	0.000 (0.00, 0.15)	1.000 (0.90, 1.00)	7.4s (4.9s,12.2s)
	LASSO	0.445 (0.23, 1.13)	1.204 (0.64, 3.07)	1.000 (0.80, 1.00)	0.000 (0.00, 0.10)	1.000 (0.95, 1.00)	12.3s (8.4s,20.5s)
Setting 3	Random	0.669 (0.21, 3.66)	1.856 (0.58, 12.96)	1.000 (0.30, 1.00)	0.000 (0.00, 0.55)	1.000 (0.75, 1.00)	15.7s (10.4s,26.6s)
	Ridge	1.019 (0.21, 3.58)	2.680 (0.62, 11.69)	0.900 (0.40, 1.00)	0.000 (0.00, 0.33)	0.999 (0.75, 1.00)	8.5s (3.0s,19.1s)
	Elas. Net.	0.423 (0.19, 1.68)	1.170 (0.53, 4.19)	1.000 (0.80, 1.00)	0.000 (0.00, 0.10)	1.000 (0.95, 1.00)	10.6s (6.0s,17.9s)
	LASSO	0.394 (0.18, 1.44)	1.118 (0.53, 3.28)	1.000 (0.90, 1.00)	0.000 (0.00, 0.09)	1.000 (0.95, 1.00)	11.8s (7.0s,19.8s)
Setting 4	Random	0.464 (0.21, 2.88)	1.289 (0.57, 9.30)	1.000 (0.60, 1.00)	0.000 (0.00, 0.33)	1.000 (0.84, 1.00)	17.1s (11.2s,27.4s)
	Ridge	0.562 (0.22, 3.08)	1.562 (0.63, 9.86)	1.000 (0.50, 1.00)	0.000 (0.00, 0.33)	1.000 (0.80, 1.00)	10.9s (4.8s,17.5s)
	Elas. Net.	0.398 (0.19, 1.41)	1.071 (0.50, 3.40)	1.000 (0.90, 1.00)	0.000 (0.00, 0.10)	1.000 (0.95, 1.00)	10.6s (6.8s,17.0s)
	LASSO	0.390 (0.18, 1.15)	1.058 (0.50, 3.05)	1.000 (0.90, 1.00)	0.000 (0.00, 0.10)	1.000 (0.95, 1.00)	12.0s (6.5s,21.3s)

Table 1: Sensitivity to different initialization methods for μ. Presented are the median and $(5\%, 95\%)$ quantiles. Data is generated taking $(n, p, s, c) = (200, 1000, 10, 0.25)$.

D.1.2 Sensitivity to σ and γ

To examine the sensitivity with respect to σ and γ we performed a grid search examining starting values of $S \times \Gamma$ where $S = \{0.01, 0.05, 0.10, 0.25, 0.5, 0.75, 1.0\}$ and $\Gamma = \{0.01, 0.05, 0.10, 0.25, 0.5, 0.75\}$. To examine the sensitivity we compute and present the mean ℓ_2-error, ℓ_1-error, TPR, FDR, AUC and runtime (in seconds) across 100 runs.

Within the simplest setting we notice the method is not sensitive to the starting values, obtaining an ℓ_2 error of 0.41 and ℓ_1-error of 1.09 and the ideal values for the TPR, FDR and AUC across the different values of σ and γ (Figure 1). More interestingly, the method can be sensitive to the initial values of σ and γ in more complicated settings (settings 2-4). Specifically, when the γ_js are small the performance is worse than when they are larger (Figures 2 - 4), for instance using a value of $\gamma_j = 0.01, j = 1, \dots, p$, would give a worse performance across all metrics in comparison to a value of $\gamma_j = 0.5$. Furthermore, within setting 2, we notice the performance is sensitive to both the values of σ and γ, with the optimal across all metrics except the FDR given by $\sigma_j = 0.1$ and $\gamma_j = 0.5$ (Figure 2). Finally, for settings 3 and 4 the method is not particularly sensitive to the value of σ, however can be sensitive to the value of γ. Therefore choosing a starting value of γ of at least 0.5 is appropriate within these settings. It is worth pointing out, that in some cases overflow issues were encountered when the values of σ_j and γ_j were too large, for instance when $\gamma_j = 0.75$ and $\sigma_j = 1$.

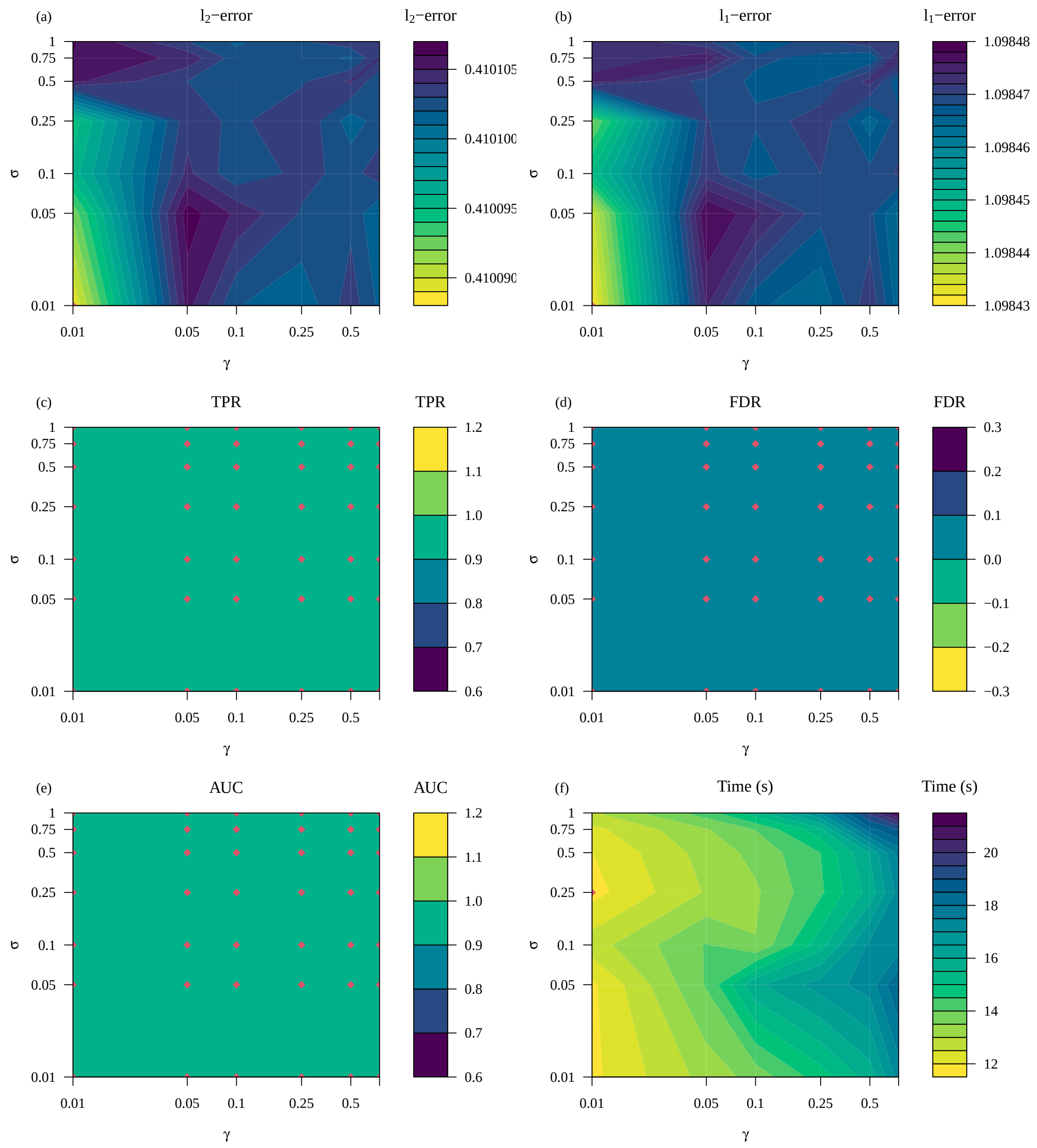

Figure 1: **Setting 1**: sensitivity with respect to σ and γ

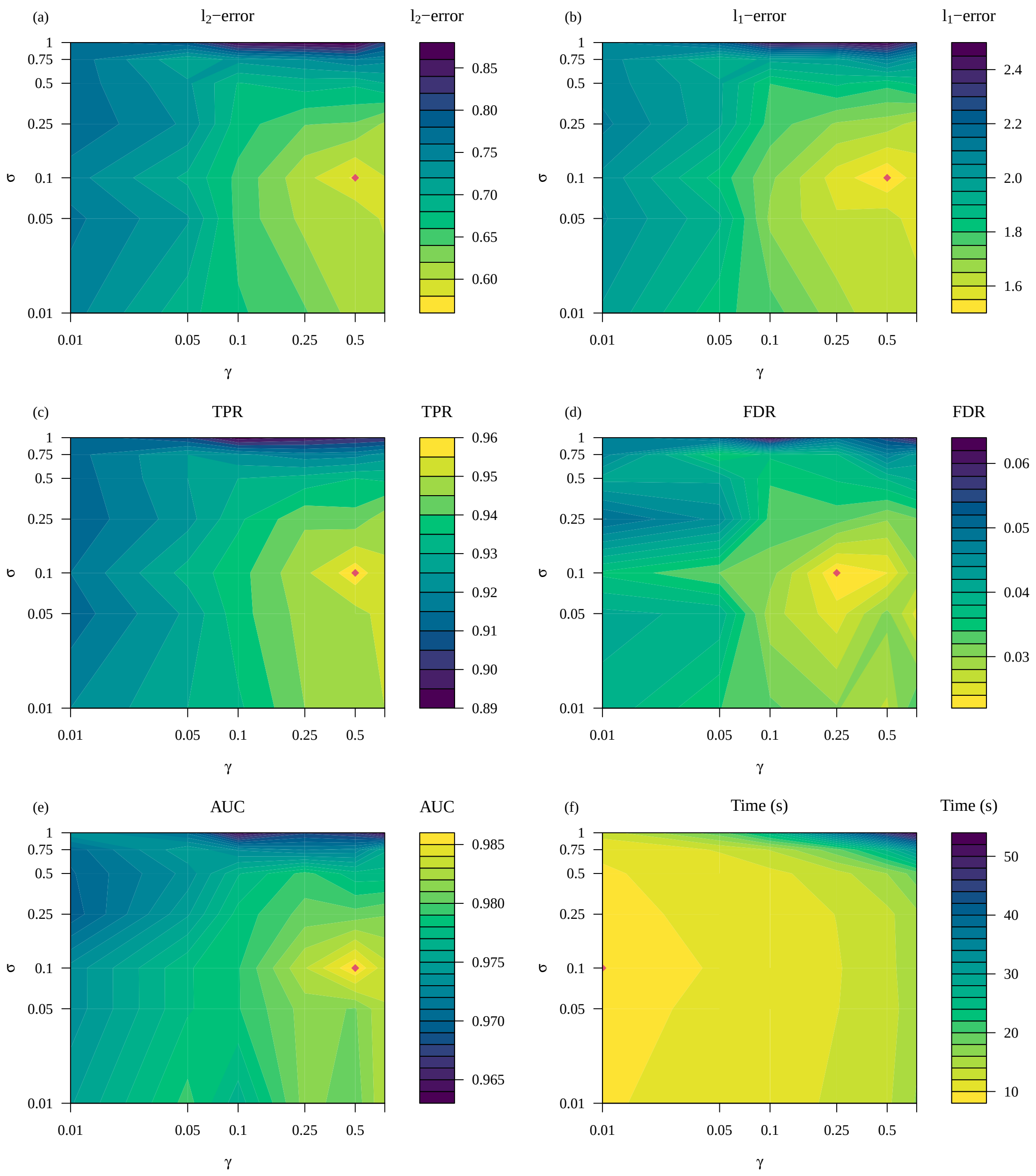

Figure 2: **Setting 2**: sensitivity with respect to σ and γ

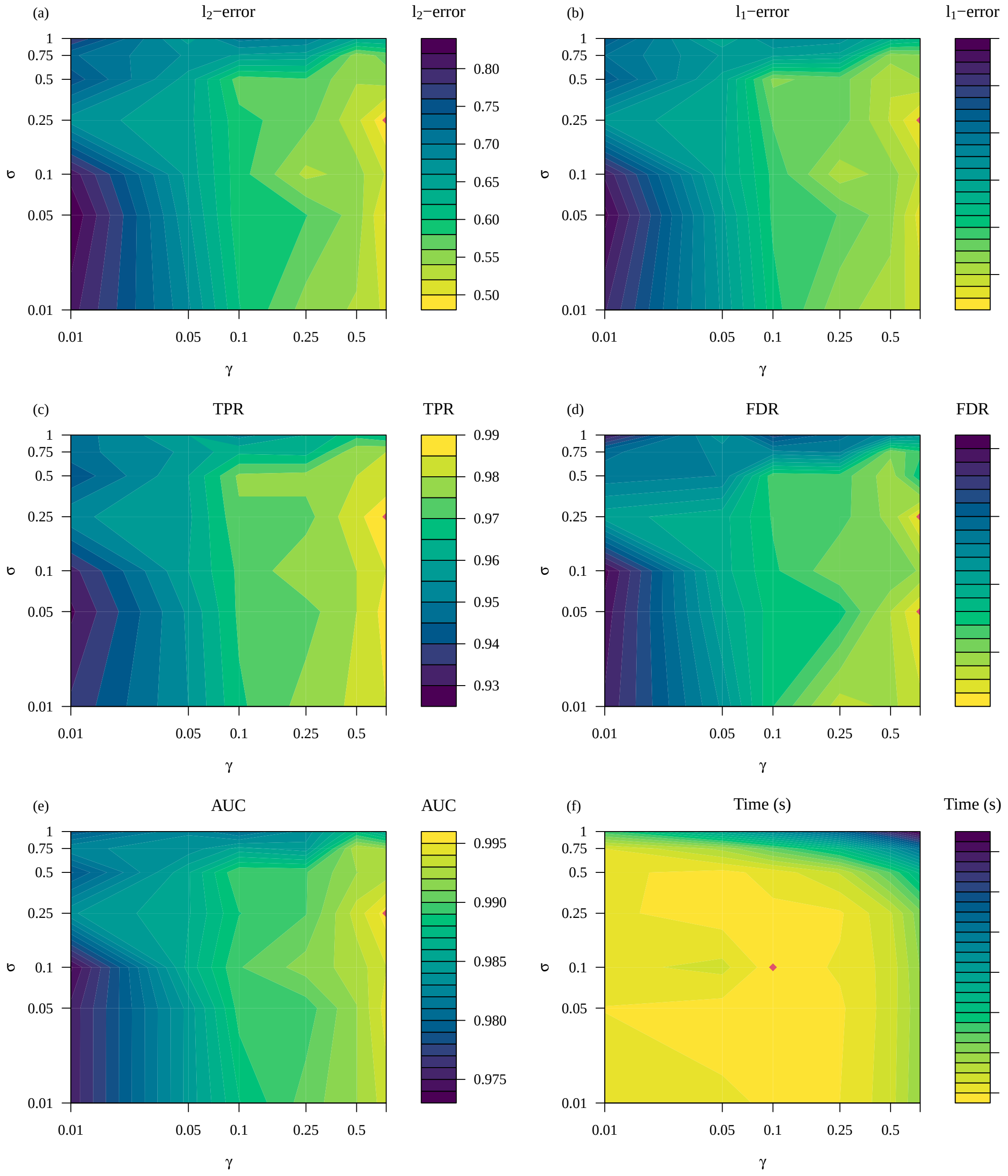

Figure 3: **Setting 3**: sensitivity with respect to σ and γ

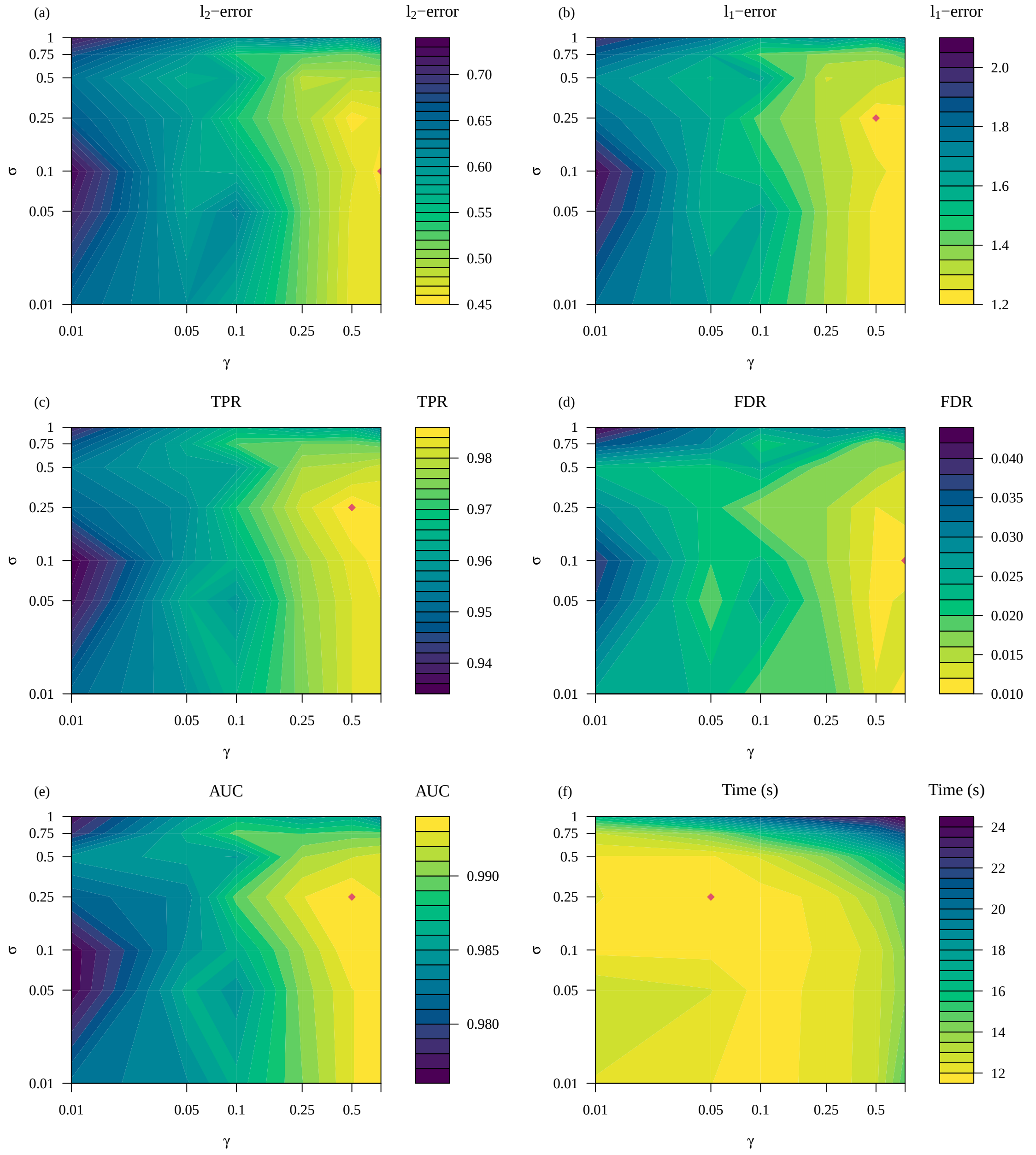

Figure 4: **Setting 4**: sensitivity with respect to σ and γ

D.2 Sensitivity to prior parameters

To evaluate the sensitivity to the prior parameters we ran simulations for Settings 1-4 taking $(n, p, s, c) = (500, 5000, 30, 0.25)$ and fit the VB posterior. To evaluate the performance we compute: (i) ℓ_2-error, (ii) ℓ_1-error, (iii) TPR, (iv) FDR, (v) AUC and (vi) runtime (ran on Intel® Xeon® E5-2680 v4 2.40GHz CPUs), reporting the mean across 100 replications. To assess the model's goodness of fit we compute the: (i) ELBO, (ii) ELL under the VB posterior, (iii) c-index, and report the mean across the 100 replications in the following figures.

D.2.1 Sensitivity to λ

We consider a grid of values $\Lambda = \{0.25, 0.5, 1, 2, 4, 8, 10, 20\}$ and fix $a_0 = 1$. For each $\lambda \in \Lambda$ we fit the VB posterior and compute the above performance metrics and goodness of fit measures, reporting the means across 100 replications in Figure 5.

Generally, the proposed method is not particularly sensitive to the value of λ and performs comparably for values between 0.25 and 2, Figure 5 (a)-(e). However, as λ increases there is an increase in the ℓ_2-error, ℓ_1-error, meaning the resulting point estimate for β_0 is being overly shrunk, i.e. high prior probability is placed about zero. Further, as λ increases above 10 there is a decrease in TPR and AUC, meaning that fewer features are correctly identified. The decrease in TPR simultaneously leads to an increase in FDR as there are proportionally more incorrectly identified features than correctly identified features. Finally, we note, as λ increases there is a decrease in runtime, Figure 5 (f).

Overall, the ELBO seems to be the most appropriate goodness of fit measure, as it is maximized for model parameter λ that yields the lowest ℓ_2-error ℓ_1-error and largest TPR. Within these settings the ELL and c-index do not identify the model with the best performance metric and in turn should be used cautiously.

D.2.2 Sensitivity to a_0

To examine the sensitivity with respect to a_0, we fix $\lambda = 1$ and examine the grid of values of $A_0 = \{1, 2, 5, 10, 20, 50, 100, 500\}$. For each $a_0 \in A_0$ we compute the VB posterior and report the respective performance metrics and goodness of fit measures (averaged across 100 replications) in Figure 6.

Generally, the proposed method is not particularly sensitive to the value of a_0, performing equivalently for values between 1 and 100, Figures 6 (a)-(e). However, for large values of a_0 the performance decreases, arising as a large value of a_0 corresponds to us believing *a priori* that many coefficients are non-zero, meaning in the resulting models many coefficients will be non-zero. This behaviour is observed in our results through the increased ℓ_1-error, ℓ_2-error and FDR. Finally, as a_0 increases the runtime increases Figure 6 (f).

Regarding goodness of fit measures, the ELBO is maximized for models with correspondingly good performance metrics. Further, the ELL and c-index increase as a_0 increases, meaning models with many non-zero parameters are favoured. These performance metrics should therefore be used cautiously when tuning a_0.

D.2.3 Sensitivity to λ and a_0

Finally, we consider the sensitivity for both parameters. To do so, we fit the VB posterior for $(\lambda, a_0) \in \Lambda \times A_0$, where Λ and A_0 are the same sets defined earlier. The results for the respective performance metrics and goodness of fit measures are presented in Figures 7 - 10 for settings 1 to 4 respectively.

Generally our method is not sensitive to the hyperparameter values in the simpler settings (1 and 2). For instance, examining Figures 7 and 8 (a) - (e), we notice that the optimal value of the TPR, FDR and AUC is attained for multiple combination of a_0 and λ. Regarding the more complicated settings (3 and 4), our method can be sensitive to λ and a_0, for instance, in setting 3, poor selection of a_0 and λ can have an impact on the FDR (Figure 9 (d)), in addition, metrics are not consistently optimal for a single combination of λ and a_0. For example, in setting 3 the optimal value of ℓ_2-error is obtained when $(\lambda, a_0) = (2, 100)$ whereas the optimal AUC is obtained when $(\lambda, a_0) = (4, 100)$, meaning in practice a trade-off may need to be made between better point estimates and variable selection.

As before, the ELBO seems to the most appropriate goodness of fit measure for most settings. However, in highly correlated settings, where there is no single "best" parameter values, the optimal ELBO corresponds the model with smallest ℓ_1-error [Figures 7 (g) and 10 (g)]. Meaning, other measures are more appropriate if the practitioner wishes to optimise other performance metrics, e.g. the TPR. Finally, the ELL and c-index should be used cautiously as these favour models with many non-zero coefficients and should therefore be used for tuning λ when a_0 is fixed.

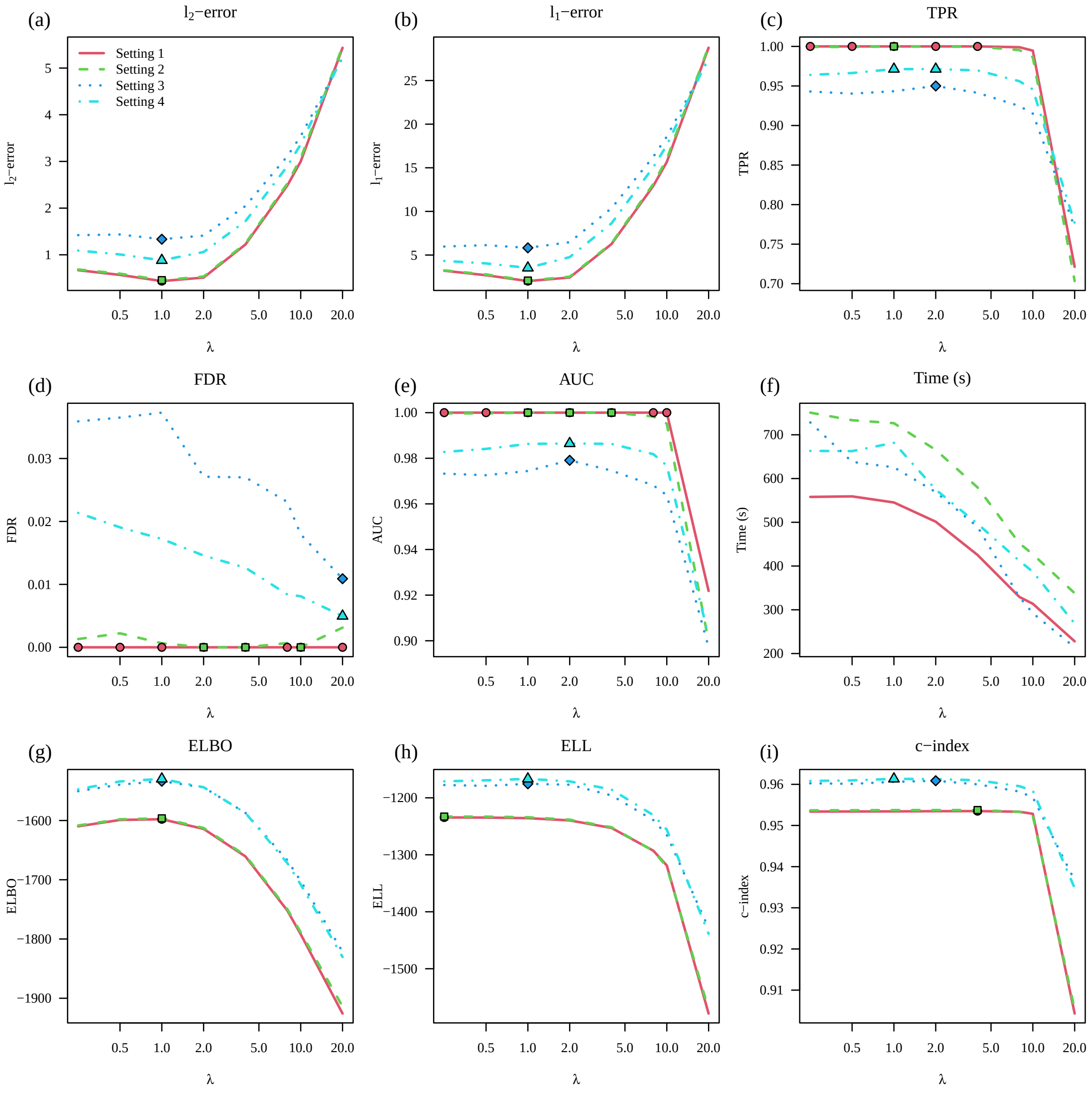

Figure 5: Sensitivity to λ for $a_0 = 1$.

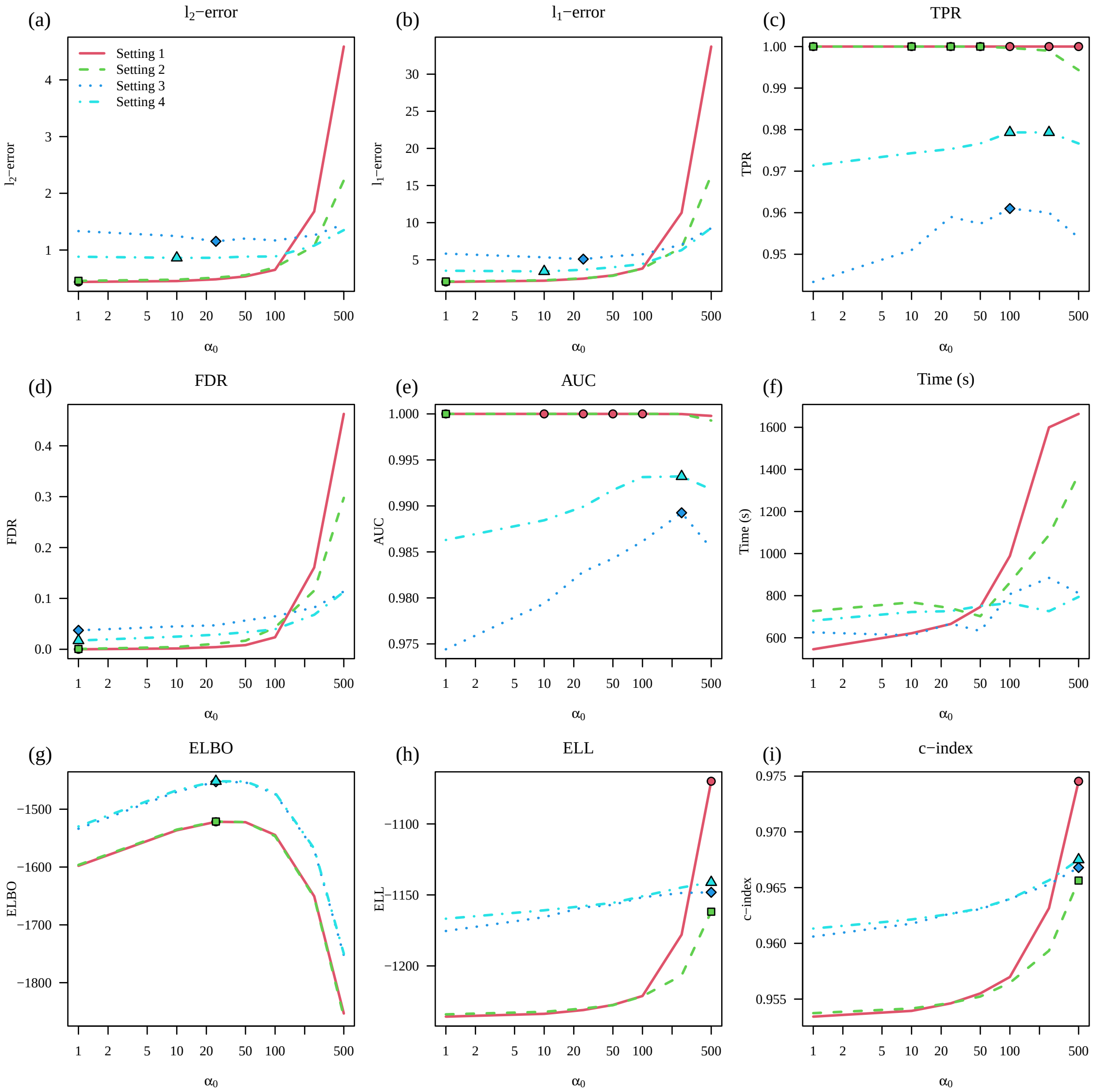

Figure 6: Sensitivity to a_0 for $\lambda = 1$

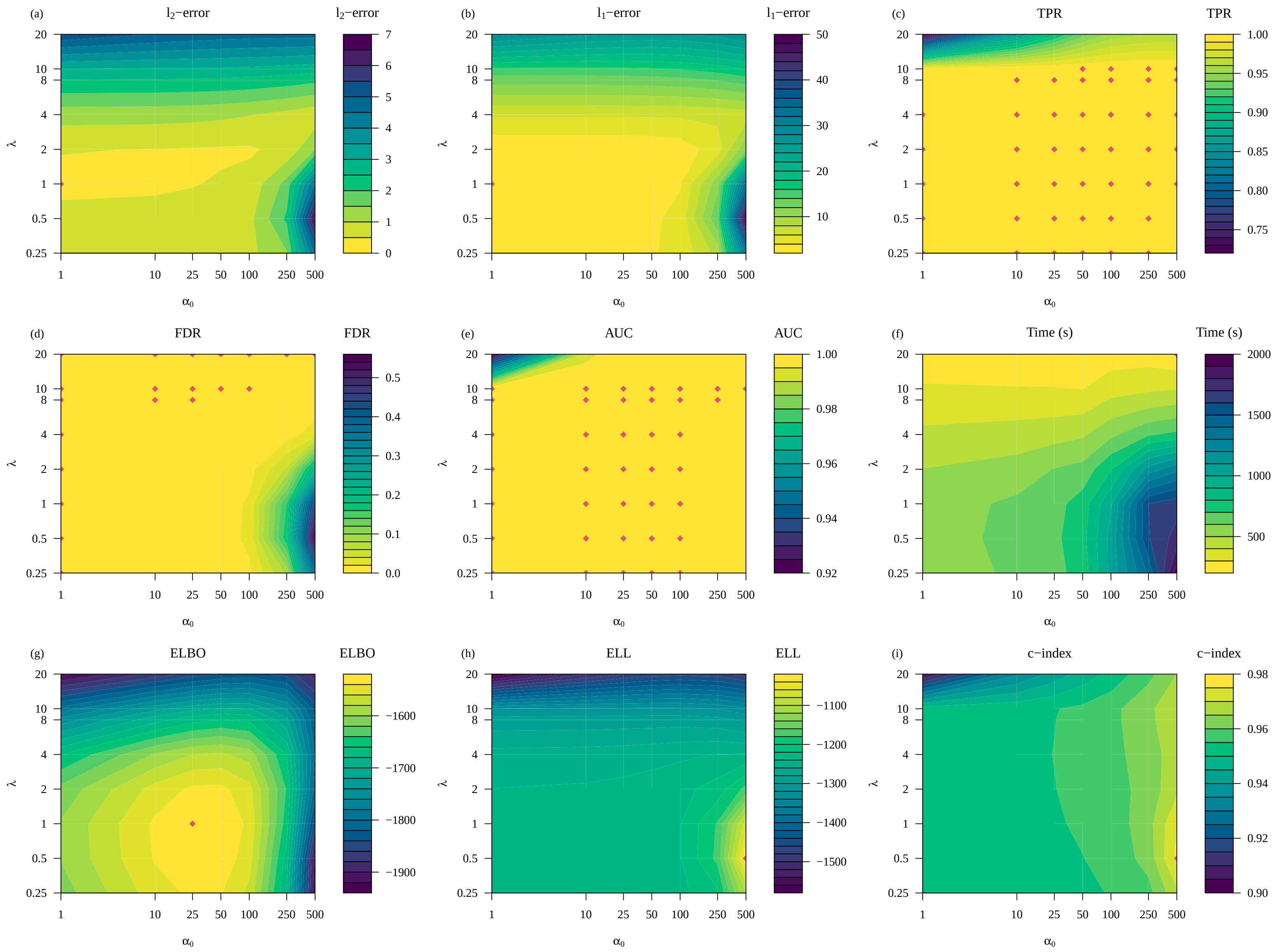

Figure 7: **Setting 1**: sensitivity with respect to λ and a_0.

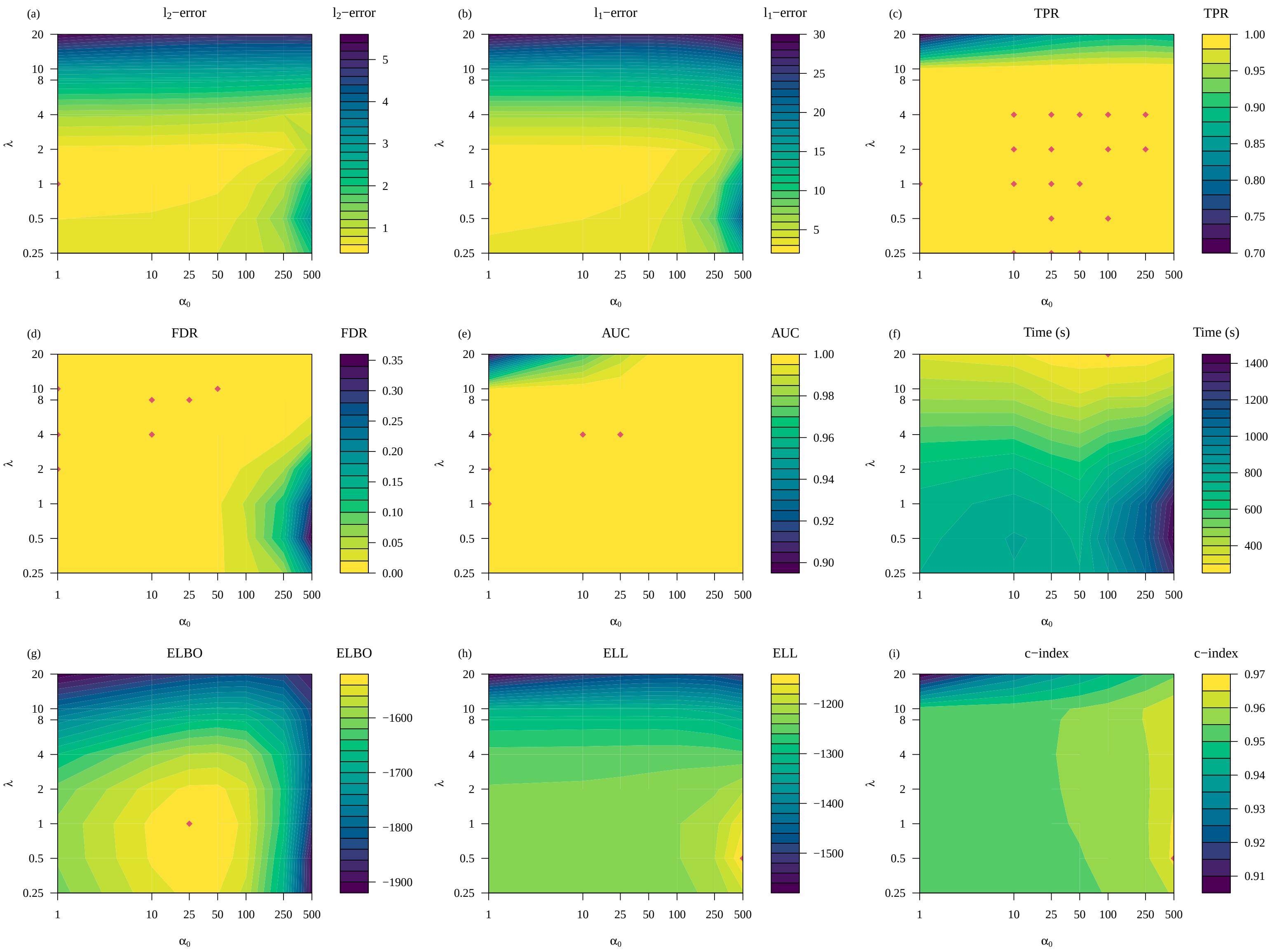

Figure 8: **Setting 2**: sensitivity with respect to λ and a_0.

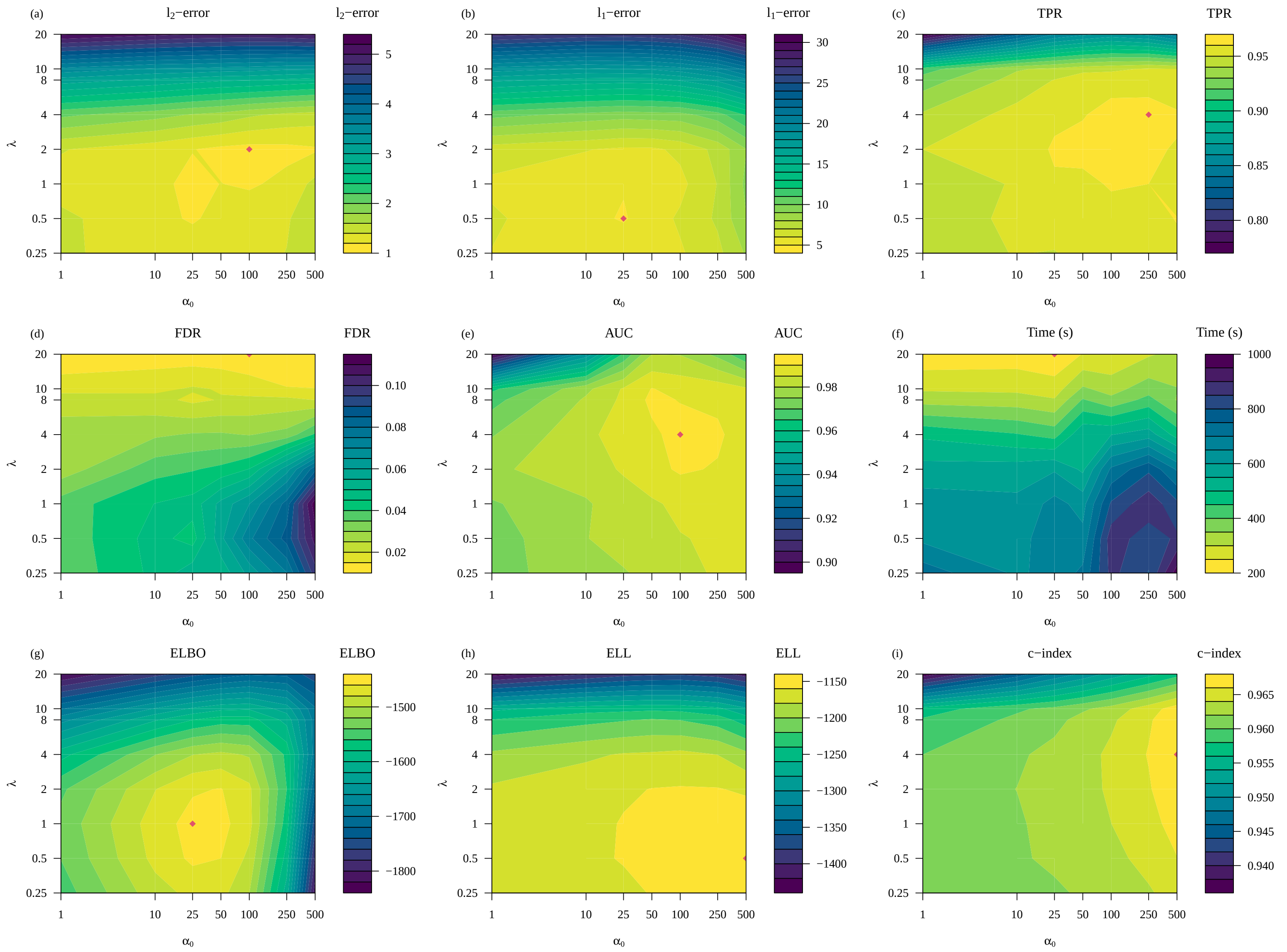

Figure 9: **Setting 3**: sensitivity with respect to λ and a_0.

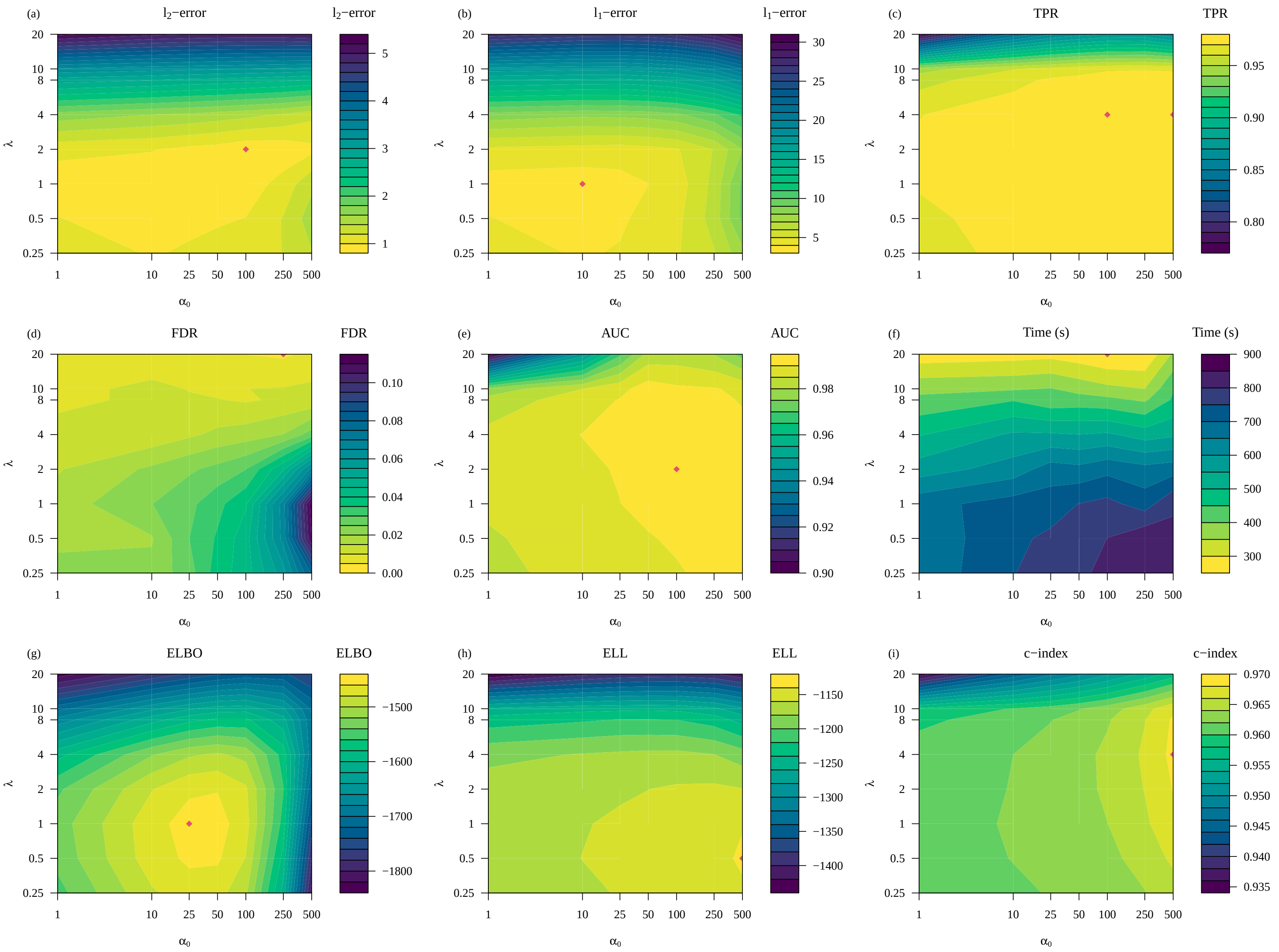

Figure 10: **Setting 4**: sensitivity with respect to λ and a_0.

E Application to real data

E.1 Availability of datasets

The datasets can be freely downloaded from:

- Ovarian cancer dataset: `https://xenabrowser.net/datapages/?cohort=GDC%20TCGA%20Ovarian%20Cancer%20(OV)&removeHub=https%3A%2F%2Fxena.treehouse.gi.ucsc.edu%3A443`
- Breast cancer dataset: `https://xenabrowser.net/datapages/?cohort=Breast%20Cancer%20(Yau%202010)&removeHub=https%3A%2F%2Fxena.treehouse.gi.ucsc.edu%3A443`

E.2 Ovarian cancer dataset

We examine the low and high risk groups constructed using the prognostic index for the ovarian cancer dataset. To construct the groups, we selected the model fit with $\lambda = 0.5$ for the first fold, which has the validation c-index of 0.57, the lowest value obtained across the different values of λ and folds. We chose this value of λ and fold to demonstrate how comparison of groups can highlight outliers and indicate when splitting based on the prognostic index may not be appropriate.

As before, to construct the groups, the validation set was split based on the median value of the prognostic index computed on the training set. The pairwise posterior probability that a patient within the high risk group is at greater risk than a patient within the low risk group is presented in Figure 11. Immediately we notice two patients within each group that may be outliers: patient 2 within the high-risk group and patient 20 within the low-risk group. In the case of patient 2 within the high-risk group, examining the row from left to right we notice the posterior probability is decreasing from around 0.8 to 0.4, meaning that on furthest end, the high-risk patients in the low-risk groups are at greater risk than patient 2. Regarding patient 20 from the low risk group, we notice the posterior probability across the column is lower in comparison to the other patients within the group. Furthermore the mean posterior probability across the column is 0.73, meaning this may be a borderline case between being in the high and low risk group.

E.3 Breast cancer dataset

Convergence diagnostics for the model fit to the breast cancer dataset are presented in Figure 12 for the ovarian and breast cancer datasets respectively. As with the TCGA data we notice the ELBO

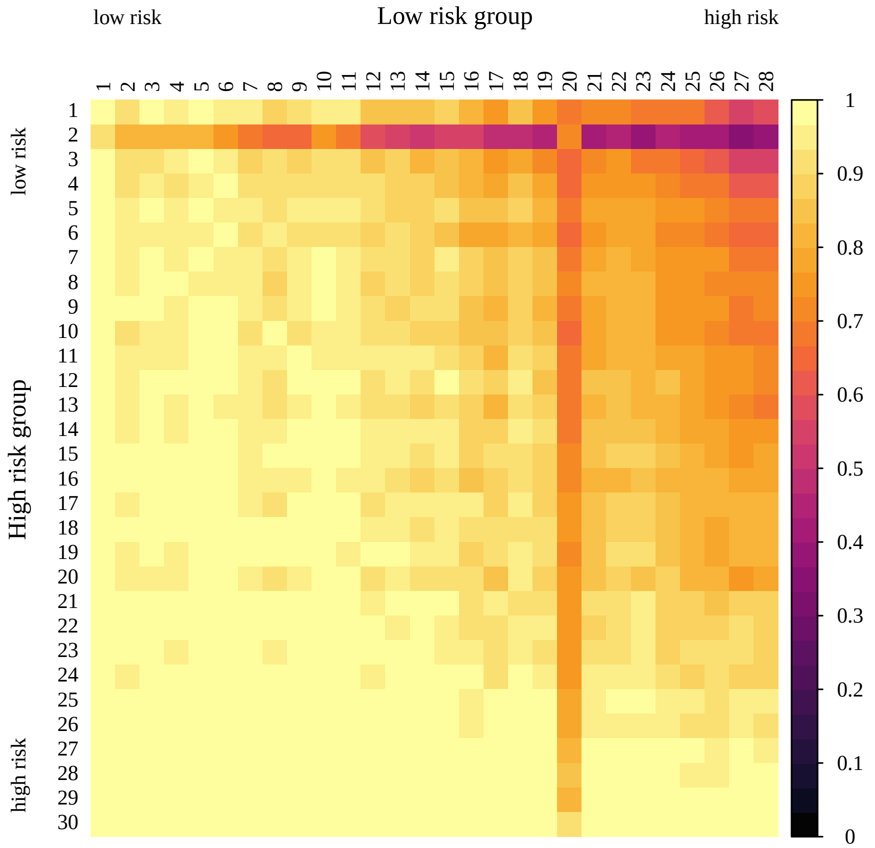

Figure 11: Comparison of validation risk groups for the TCGA ovarian cancer dataset. Rows correspond to patients in the high-risk group and columns to patients in the low-risk group. Both rows and columns are sorted from lowest to highest risk.

is increasing as we iterate the algorithm, suggesting the model fit is improving. Furthermore, we notice that the $\text{KL}(Q\|\Pi)$ is decreasing, suggesting that sparsity is induced. Coupled with the fact the validation expected log-likelihood is increasing, this suggests fewer spurious variables are being selected and the model is fitting better to unseen validation set.

E.4 Model fits

Model fit for different values of λ for the two datasets are presented in Table 2 and Table 3. Reported is the mean and standard deviation across 10 training and validation folds of the: ELBO, ELL, KL, c-index $(\widehat{k})$ and the number of parameters for which $\gamma_j > 0.5$. Examining the different metrics, we notice the model is not particularly sensitivity to the value of λ.

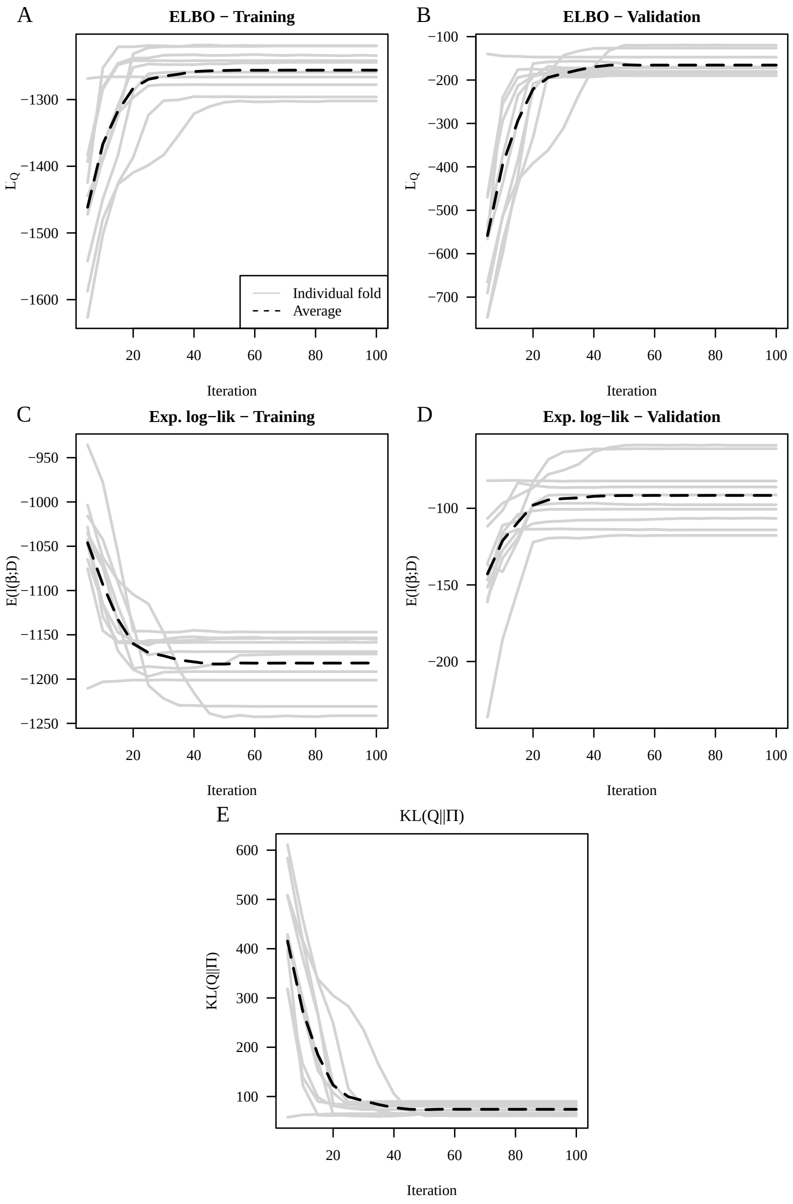

Figure 12: Breast cancer dataset convergence diagnostics for model is fit with $\lambda = 2.5$, Presented is the: ELBO, ELL and KL for the training and validation set. Each fold is presented in (solid) grey, and the mean over the 10 folds presented in (dashed) black. As with the TCGA, we notice as we iterate the model fit improves, fitting better to the unseen validation set.

λ	Training					Validation			
	ELBO	ELL	KL	$\widehat{k}$	$\#\{\gamma > 0.5\}$	ELBO	ELL	KL	$\widehat{k}$
0.05	-1735.7 (21.2)	-1657.5 (26.1)	78.2 (12.2)	0.628 (0.018)	2.0 (1.4)	-189.3 (20.1)	-111.1 (12.3)	78.2 (12.2)	0.558 (0.044)
0.10	-1733.7 (20.6)	-1647.0 (31.9)	86.8 (13.7)	0.646 (0.018)	3.1 (1.7)	-197.8 (24.5)	-111.0 (12.3)	86.8 (13.7)	0.572 (0.041)
0.25	-1795.2 (208.5)	-1604.1 (129.5)	191.1 (333.6)	0.687 (0.085)	15.9 (40.5)	-322.2 (398.1)	-131.1 (65.3)	191.1 (333.6)	0.581 (0.042)
0.50	-1725.8 (21.3)	-1635.1 (30.9)	90.7 (15.1)	0.681 (0.021)	4.3 (2.1)	-202.3 (25.1)	-111.7 (13.0)	90.7 (15.1)	0.588 (0.030)
0.75	-1721.8 (22.4)	-1631.3 (31.2)	90.5 (13.4)	0.695 (0.015)	4.5 (2.0)	-202.8 (24.5)	-112.3 (14.1)	90.5 (13.4)	0.590 (0.050)
1.00	-1719.2 (20.6)	-1620.4 (37.5)	98.8 (26.8)	0.701 (0.023)	6.3 (3.9)	-211.6 (37.3)	-112.8 (13.6)	98.8 (26.8)	0.585 (0.034)
1.25	-1713.7 (22.9)	-1622.7 (27.3)	91.0 (14.1)	0.703 (0.016)	5.5 (2.2)	-204.7 (21.0)	-113.7 (14.0)	91.0 (14.1)	0.594 (0.037)
1.50	-1712.8 (22.9)	-1620.3 (28.4)	92.4 (15.0)	0.706 (0.015)	6.1 (2.4)	-205.4 (24.8)	-113.0 (14.5)	92.4 (15.0)	0.587 (0.043)
1.75	-1709.9 (20.8)	-1625.1 (29.9)	84.9 (12.0)	0.704 (0.014)	5.2 (1.9)	-197.7 (24.0)	-112.8 (14.5)	84.9 (12.0)	0.605 (0.044)
2.00	-1707.6 (21.7)	-1618.2 (28.5)	89.4 (13.1)	0.717 (0.021)	6.1 (2.0)	-202.8 (22.7)	-113.4 (14.1)	89.4 (13.1)	0.589 (0.038)
2.50	-1704.5 (22.9)	-1620.3 (28.7)	84.2 (9.1)	0.717 (0.011)	5.8 (1.5)	-198.1 (22.7)	-113.9 (16.1)	84.2 (9.1)	0.610 (0.054)
3.00	-1702.5 (21.7)	-1623.2 (31.2)	79.3 (10.6)	0.715 (0.014)	5.5 (1.8)	-191.2 (23.7)	-111.9 (13.7)	79.3 (10.6)	0.606 (0.034)
4.00	-1695.7 (21.9)	-1626.0 (28.8)	69.6 (10.7)	0.713 (0.011)	4.8 (1.8)	-181.4 (20.5)	-111.7 (13.2)	69.6 (10.7)	0.602 (0.021)
5.00	-1693.9 (21.4)	-1629.1 (25.9)	64.7 (8.7)	0.713 (0.012)	4.4 (1.3)	-176.3 (19.4)	-111.5 (13.6)	64.7 (8.7)	0.605 (0.045)

Table 2: **Ovarian cancer dataset**, model fit for different values of λ

λ	Training					Validation			
	ELBO	ELL	KL	$\widehat{k}$	$\#\{\gamma > 0.5\}$	ELBO	ELL	KL	$\widehat{k}$
0.05	-1284.1 (29.8)	-1216.9 (28.9)	67.1 (1.6)	0.693 (0.009)	2.0 (0.0)	-155.1 (18.1)	-87.9 (19.0)	67.1 (1.6)	0.625 (0.039)
0.10	-1282.2 (29.8)	-1210.6 (30.9)	71.6 (7.3)	0.706 (0.010)	2.6 (1.0)	-161.1 (22.9)	-89.5 (20.2)	71.6 (7.3)	0.615 (0.071)
0.25	-1278.9 (29.5)	-1199.7 (31.2)	79.3 (12.3)	0.729 (0.019)	4.1 (1.8)	-170.6 (24.7)	-91.4 (19.7)	79.3 (12.3)	0.598 (0.071)
0.50	-1274.6 (29.1)	-1189.3 (34.9)	85.3 (14.9)	0.746 (0.013)	5.4 (2.1)	-176.8 (29.6)	-91.6 (20.8)	85.3 (14.9)	0.615 (0.075)
0.75	-1270.2 (29.2)	-1189.0 (29.8)	81.2 (9.4)	0.752 (0.011)	5.1 (1.6)	-174.1 (22.9)	-92.9 (20.4)	81.2 (9.4)	0.593 (0.081)
1.00	-1268.4 (30.5)	-1187.8 (28.5)	80.6 (12.0)	0.760 (0.012)	5.2 (2.1)	-173.8 (22.5)	-93.2 (21.0)	80.6 (12.0)	0.603 (0.083)
1.25	-1264.1 (29.2)	-1184.0 (30.8)	80.0 (11.3)	0.768 (0.009)	5.4 (2.0)	-173.8 (24.2)	-93.8 (20.3)	80.0 (11.3)	0.590 (0.075)
1.50	-1262.9 (29.6)	-1181.5 (32.4)	81.3 (11.0)	0.772 (0.010)	5.8 (1.8)	-174.8 (25.4)	-93.4 (20.8)	81.3 (11.0)	0.596 (0.064)
1.75	-1260.9 (29.4)	-1179.0 (32.6)	81.9 (15.9)	0.776 (0.012)	6.2 (2.7)	-175.8 (27.6)	-93.9 (20.6)	81.9 (15.9)	0.597 (0.064)
2.00	-1259.1 (29.5)	-1180.6 (32.6)	78.5 (11.0)	0.775 (0.009)	5.7 (1.8)	-172.6 (25.0)	-94.1 (20.5)	78.5 (11.0)	0.584 (0.077)
2.50	-1255.9 (29.5)	-1181.9 (33.3)	74.0 (10.1)	0.777 (0.009)	5.3 (1.6)	-165.6 (25.5)	-91.6 (20.1)	74.0 (10.1)	0.626 (0.075)
3.00	-1252.8 (29.5)	-1184.1 (30.1)	68.7 (8.7)	0.781 (0.005)	4.8 (1.6)	-161.6 (22.0)	-92.9 (19.6)	68.7 (8.7)	0.612 (0.072)
4.00	-1249.3 (29.7)	-1183.1 (31.1)	66.2 (7.7)	0.785 (0.004)	4.9 (1.4)	-158.6 (23.1)	-92.4 (20.2)	66.2 (7.7)	0.619 (0.072)
5.00	-1246.6 (28.7)	-1185.8 (31.4)	60.8 (9.4)	0.787 (0.008)	4.5 (1.8)	-153.2 (24.8)	-92.4 (20.2)	60.8 (9.4)	0.610 (0.069)

Table 3: **Breast cancer dataset**, model fit for different values of λ

References

Christopher M. Bishop. *Pattern Recognition and Machine Learning*. Springer, 2006.

Mithat Gonen and Glenn Heller. Concordance probability and discriminatory power in proportional hazards regression. *Biometrika*, 92(4):965–970, 2005.

Jr Harrell, Frank E., Robert M. Califf, David B. Pryor, Kerry L. Lee, and Robert A. Rosati. Evaluating the yield of medical tests. *JAMA*, 247(18):2543–2546, 05 1982. ISSN 0098-7484. doi: 10.1001/jama.1982.03320430047030. URL https://doi.org/10.1001/jama.1982.03320430047030.

David J. Nott, Siew Li Tan, Mattias Villan, and Robert Kohn. Regression density estimation with variational methods and stochastic approximation. *Journal of Computational and Graphical Statistics*, 21(3):797–820, 2012. ISSN 10618600. doi: 10.1080/10618600.2012.679897.
